# Bioactivity of Spongian Diterpenoid Scaffolds from the Antarctic Sponge *Dendrilla antarctica*

**DOI:** 10.3390/md18060327

**Published:** 2020-06-23

**Authors:** Alexandre Bory, Andrew J. Shilling, Jessie Allen, Ala Azhari, Alison Roth, Lindsey N. Shaw, Dennis E. Kyle, John H. Adams, Charles D. Amsler, James B. McClintock, Bill J. Baker

**Affiliations:** 1 Department of Chemistry, University of South Florida, 4202 E. Fowler Avenue, CHE205, Tampa, FL 33620, USA; alexandre.bory@pharma.ethz.ch (A.B.); ashillin@usf.edu (A.J.S.); 2 Department of Cell Biology, Microbiology, and Molecular Biology, University of South Florida, 4202 E. Fowler Avenue, ISA2015, Tampa, FL 33620, USA; jessiea@usf.edu (J.A.); shaw@usf.edu (L.N.S.); 3 USF Center for Global Health and Infectious Diseases Research, University of South Florida, 3010 USF Banyan Circle, IDRB 304, Tampa, FL 33612, USA; aaazhari@kau.edu.sa (A.A.); aroth1@usf.edu (A.R.); dennis.kyle@uga.edu (D.E.K.); ja2@usf.edu (J.H.A.); 4 Department of Biology, University of Alabama at Birmingham, Birmingham, AL 35294, USA; amsler@uab.edu (C.D.A.); mcclinto@uab.edu (J.B.M.)

**Keywords:** diterpenoids, dendrillins, malaria, leishmaniasis, MRSA biofilm

## Abstract

The Antarctic sponge *Dendrilla antarctica* is rich in defensive terpenoids with promising antimicrobial potential. Investigation of this demosponge has resulted in the generation of a small chemical library containing diterpenoid secondary metabolites with bioactivity in an infectious disease screening campaign focused on *Leishmania donovani*, *Plasmodium falciparum*, and methicillin-resistant *Staphylococcus aureus* (MRSA) biofilm. In total, eleven natural products were isolated, including three new compounds designated dendrillins B–D (**10**–**12**). Chemical modification of abundant natural products led to three semisynthetic derivatives (**13**–**15**), which were also screened. Several compounds showed potency against the leishmaniasis parasite, with the natural products tetrahydroaplysulphurin-1 (**4**) and dendrillin B (**10**), as well as the semisynthetic triol **15**, displaying single-digit micromolar activity and low mammalian cytotoxicity. Triol **15** displayed the best profile against the liver-stage malaria parasites, while membranolide (**5**) and dendrillin C (**11**) were strong hits against MRSA biofilm cultures.

## 1. Introduction

As the pharmaceutical industry has deprioritized research into infectious disease treatments in recent decades, new antimicrobial scaffolds are now being discovered less frequently [1,2]. The recent lull in novel antimicrobial development has steadily reduced the number of effective first-line treatments available, resulting in the gradual increase of drug-resistant infections, with over 2.8 million cases reported in the United States in 2019 [3]. The severity of this problem is predicted to escalate exponentially as resistance continues to surge, with the global death toll attributed to antimicrobial resistant infections estimated to exceed 10 million by the year 2050 [4]. Infectious parasitic diseases carry a significant global disease burden [5] with malaria and leishmania alone contributing to nearly half a million annual deaths. The need for the development of novel classes of infectious disease treatments is critical to the continued advancement of modern medicine [2,6,7].

Natural products have historically provided many anti-infective drugs in our pharmacopeia [8]. Among marine sources of biodiscovery leads, sponges are the most chemically rich invertebrates [9], yielding several promising candidates for drug development [10]. The majority of these drug leads have historically come from sponges found in areas with warm or temperate climates, while the polar regions are underrepresented, mostly owing to issues with accessibility and difficulties working in the harsh conditions common to these environments. However, the past few decades have seen a shift in the focus of biodiscovery towards the lesser explored cold waters of the Arctic and Antarctica, and the sponges that dwell there could potentially represent a new source of treatments against infectious diseases [11,12,13].

**Figure 1:**
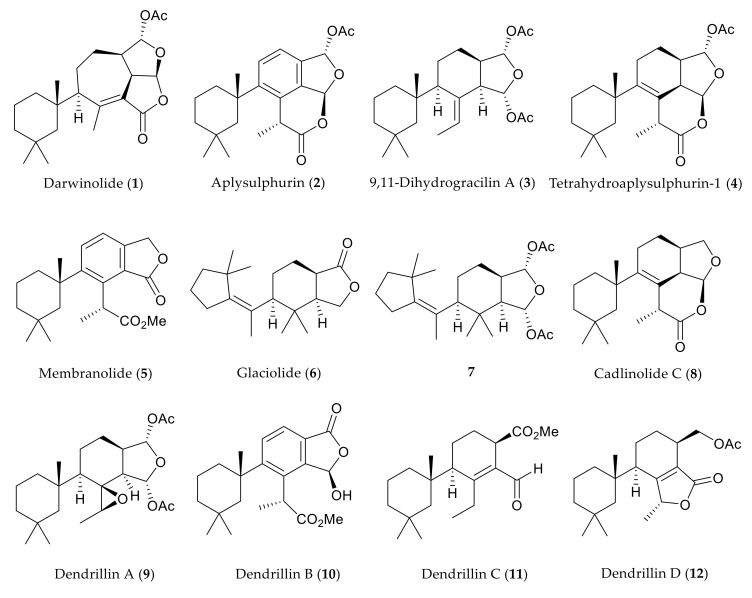
Suite of *Dendrilla antarctica* metabolites from previous (**1**–**9**) and current (**10**–**12**) reports.

Previously, we have reported cold-water marine natural products with activity against leishmaniasis [14,15], malaria [16], and pathogenic bacteria [17]. The Antarctic sponge *Dendrilla antarctica* (previously *D. membranosa*) is a prolific source of highly oxidized diterpenoids, including methyl acetals isolated from methanolic extracts, which displayed moderate antibiotic and antifungal activities against *Staphylococcus aureus*, *Escherichia coli*, and *Candida albicans*, respectively [18]. Subsequent chemical analysis of *D. antarctica* yielded a new diterpenoid darwinolide (**1**), bearing a unique seven-membered ring rearranged diterpene skeleton (Figure 1). This new natural product shows promise as a developmental scaffold for methicillin-resistant *Staphylococcus aureus* (MRSA) biofilm treatment, as it was four times more potent against the biofilm than it was against planktonic MRSA, with relatively low mammalian cytotoxicity [17]. This type of selective toxicity towards biofilms is notable owing to the lack of current antibiofilm-specific antibiotics. Recent collections of *D. antarctica* from the vicinity of the U.S. Antarctic research base at Palmer Station, Antarctica, in the Austral summers of 2016–2018 have resulted in eight known (**2**–**9**) [19,20,21,22,23,24,25] and three new metabolites, dendrillins B–D (**10**–**12**). To further explore the bioactivity of the *D. antarctica* spongian scaffolds, three semisynthetic derivatives were prepared from the major metabolites.

## 2. Results and Discussion

### 2.1. Chemical Analysis of Dendrilla antarctica

Collections of *Dendrilla antarctica* were made in three consecutive field seasons, March–June 2016, 2017, and 2018. From the 2016 collection, freeze-dried sponge was extracted with dichloromethane. Purification of the concentrated extract by normal phase medium pressure liquid chromatography (MPLC) afforded 13 fractions, with one containing pure aplysulphurin (**2**). Other fractions were further purified by HPLC to give known natural products 9,11-dihydrogracilin A (**3**), tetrahydroaplysulphurin-1 (**4**), membranolide (**5**), and the new minor metabolite dendrillin B (**10**). The subsequent collections from 2017 and 2018 were extracted and fractionated using the same protocol, resulting in minor constituents glaciolide (**6**), a norditerpene **7**, dendrillin A (**9**), and previously undescribed dendrillin C (**11**) and D (**12**). Previously reported compounds were characterized based on comparison of their NMR spectra to the published values. Dendrillins B–D are described here.

**Figure 2:**
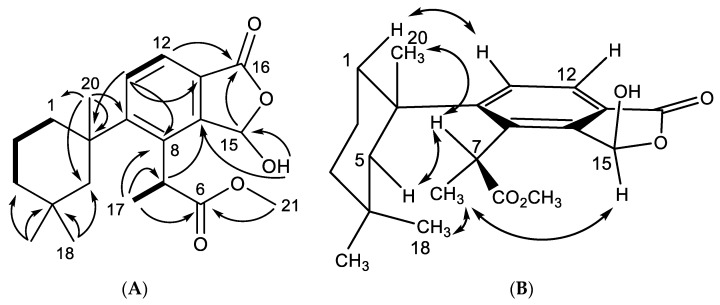
Key (**A**) HMBC (→), COSY (▬), and (**B**) NOESY (↔) correlations establishing the relative configuration for dendrillin B (**10**).

Dendrillin B (**10**) was obtained as a clear oil with spectral data similar to that of previously reported membranolide (**5**), but with differences among the NMR signals of key functional groups about the aromatic ring. A formula of C_21_H_28_O_5_ was established from HRESIMS. Key ^1^H NMR signals (Table 1), which established similarities to membranolide including *gem* methyl groups H_3_-18 (δ_H_ 0.36) and H_3_-19 (δ_H_ 0.93), and a singlet methyl H_3_-20 at δ_H_ 1.40 (Figure 2). H_3_-18 and H_3_-19 correlated in the HMBC spectrum to alkyl carbons C-5 (δ_C_ 51.1) and C-3 (δ_C_ 39.3). The HMBC correlation of H_3_-20 to C-5, taken with COSY correlations among H_2_-3 (δ_H_ 1.32), H-2α (δ_H_ 1.70), H-2β (δ_H_ 1.79), H-1α (δ_H_ 2.35), and H-1β (δ_H_ 1.50), defined the 4,4,10-trimethylcyclohexyl group familiar to membranolide. HMBC correlation of H_3_-20 to C-9 (δ_C_ 154.4) placed the trimethylcyclohexyl group on an aromatic ring defined by a pair of nearly overlapping aromatic protons shifted considerably downfield (H-11, δ_H_ 7.78) and (H-12, δ_H_ 7.76) and three quaternary carbons, C-8 (δ_C_ 136.6), C-13 (δ_C_ 125.9), and C-14 (δ_C_ 145.5). The lowfield position of the aromatic protons were indicative of conjugation, supported by an HMBC correlation of H-12 to carbonyl C-16 (δ_C_ 168.1). C-16 was found to be a carboxylate ester situated in a fused hydroxybenzofuranone ring system via an HMBC correlation from H-15 (δ_H_ 6.70) to C-16, of which H-15 is revealed to be a hemiacetal methine through an HMBC correlation between OH (δ_H_ 4.28) and C-15 (δ_C_ 97.3), as well as observation of coupling between H-15 and the hydroxy proton. The final substituent on the aromatic ring was found to be a methyl propionate ester ortho to the trimethylcyclohexyl group based on HMBC correlations from a doublet methyl H_3_-17 (δ_H_ 1.75; *J* = 7.5) to C-8 (δ_C_ 136.6) and C-6 (δ_C_ 176.6), which coupled to a quartet methine H-7 (δ_H_ 4.78, *J* = 7.3). H-7 displayed further HMBC correlations to C-9 and C-14 (δ_C_ 145.5). The final confirmation of the position of the terminal methyl ester is supported by an HMBC correlation from H_3_-21 (δ_H_ 3.67) to C-6 (Figure 2).

The relative configuration of dendrillin B (**10**) was established as shown in Figure 2, and is predicated on the lack of rotation about C-7 of the propionate ester owing to the steric hindrance from its close proximity to the trimethylcyclohexyl substituent [18]. The aromatic ring is expected to be in the axial position, consistent with other aromatic members of this series, including aplysulphurin [21] and membranoid A [15] (= membranolide B [18]). In this position, H_3_-18 is found in the anisotropic shielding zone of the aromatic ring, exemplified by its highfield shift (δ_H_ 0.36), and placing it in proximity of H_3_-17 to observe an NOE between the two methyl groups. Additional NOEs observed for the trimethylcyclohexane ring are consistent with membranolide (**5**) and membranoid A [15,18]. The final stereocenter about C-15 was established with the hemiacetal methine in the α-orientation owing to a NOESY correlation between H-15 (δ_H_ 6.70) and H_3_-17.

**Figure 3:**
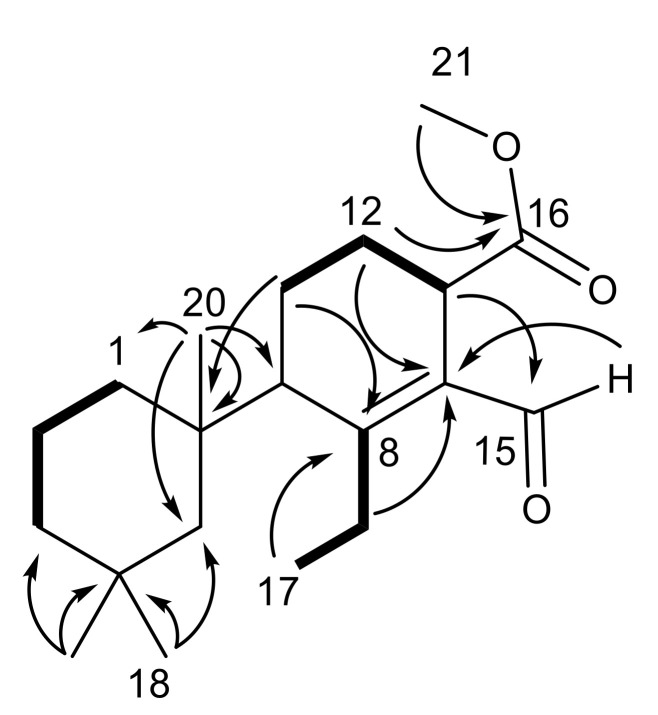
Key HMBC (→) and COSY (▬) establishing the planar structure of dendrillin C (**11**).

Dendrillin C (**11**) was obtained as a clear oil and a formula of C_20_H_32_O_3_ was established from HRESIMS. The same trimethylcyclohexane ring was evident from the H_3_-18 (δ_H_ 0.98), H_3_-19 (δ_H_ 0.92), and H_3_-20 (δ_H_ 1.03) shifts (Table 1) and corresponding HMBC correlations (Figure 3). An additional triplet methyl group H_3_-17 (δ_H_ 1.13) was shown through COSY correlation to H_2_-7 (H-7a (δ_H_ 2.43) and H-7b (δ_H_ 3.20)). That C-7 (δ_C_ 26.7) bears diastereotopic protons supports previous assignments (above) of restricted rotation in that congested ring system. H_3_-17 correlated by HMBC to C-8 (δ_C_ 167.9), and H-7b to C-14 (δ_C_ 134.2), fixing the ethyl group at C-8. Further HMBC correlations of H-11β (δ_H_ 2.01) to C-8 and H-12β (δ_H_ 1.91) to C-14, as well as a COSY correlations between H-11β and H-12β and H-12β to H-13 (δ_H_ 3.60), secured the assignment of the reduced aromatic ring that was found in dendrillin B (**10**). The presence of an aldehyde at C-15 (δ_C_ 190.5) conjugated with the Δ^8,14^ olefin was established by observation of HMBC correlation of H-15 (δ_H_ 10.18) to C-14. A methyl ester was revealed at C-16 (δ_C_ 174.8) and C-21 (δ_C_ 51.8), which could be positioned about the ring based on an HMBC correlation between H-12β and C-16.

**Figure 4:**
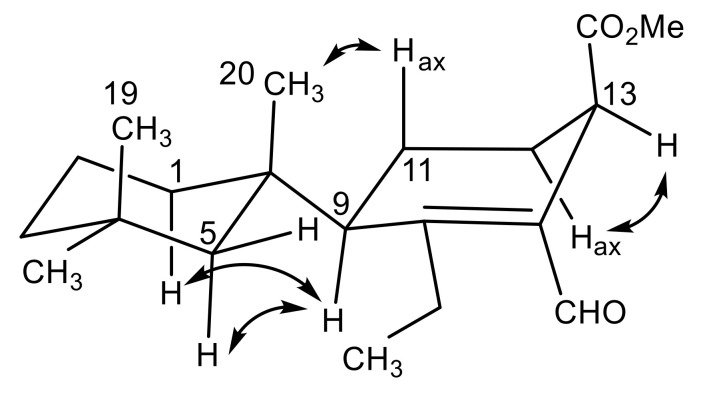
NOESY analysis establishing the relative conformation of dendrillin C (**11**).

The relative configuration of dendrillin C (**11**) was approached by comparison with the other members of this family. Two distinguishing features characterize the planar structure of **11** than influence its conformation; one is the reduced (tetrahydro) central ring and the second is the methine at C-9. Aromatic central ring members of this family of *D. antarctica* metabolites have a well-characterized configuration with the central ring substituent occupying the axial position of the trimethylcyclohexane chair conformation. This is borne out both by X-ray analysis of, for example, aplysulphurin [26], membranoids C and E [15], and darwinolide [17], and the respective upfield shift of H_3_-19 (δ_H_ ~0.5) owing to its position in the anisotropic cone of the aromatic ring. H_3_-20, in the equatorial position, similarly has a characteristic deshielded shift (~δ_H_ 1.4). Dendrillin C lacks the shielded H_3_-19 (δ_H_ 0.92) and deshielded H_3_-20 (δ_H_ 1.03) indicative of its central ring in an equatorial position on the trimethylcyclohexane ring system (Figure 4). NOESY correlations from the axial methyl H_3_-20 to equatorial H-5β (δ_H_ 1.46) and axial H_3_-19 and from equatorial H_3_-18 (δ_H_ 0.98) to axial H-5α (δ_H_ 1.35), support the conformation of the trimethylcyclohexane system illustrated in Figure 4. H-7a (δ_H_ 2.43) has a NOESY correlation to H-5β, fixing the ethyl group in the proximity of C-5. The configuration at C-13 was established with the methyl ester in the β-orientation based on a NOESY correlation between H-13 (δ_H_ 3.60) and H-12α (δ_H_ 1.82), placing the H-13 methine in the pseudoequatorial orientation.

**Figure 5:**
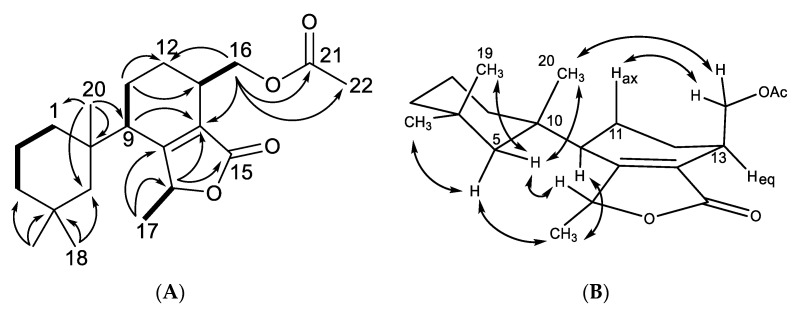
Key (**A**) HMBC (→), COSY (▬), and (**B**) NOESY (↔) correlations establishing the relative configuration for dendrillin D (**12**).

Dendrillin D (**12**) was obtained as a clear oil with spectral data unlike that of the previously isolated dendrillins and related spongian diterpenes [27], but with certain motifs conserved, including the trimethylcyclohexane substituent and its attached reduced (tetrahydro) aromatic ring. A formula of C_21_H_32_O_4_ was established from HRESIMS. Key ^1^H NMR signals (Table 1) established the presence of a C-9 (δ_H_ 2.18) methine, which displayed a COSY correlation to H_2_-11 (δ_H_ 1.74), as well as HMBC correlation to C-14 (δ_C_ 128.0). Further HMBC correlations were observed between H_2_-11/C-12 (δ_C_ 27.3) and H_2_-11/C-13 (δ_C_ 30.7), from a doublet methyl H_3_-17 (δ_H_ 1.45, *J* = 6.5) to C-8 (δ_C_ 169.3), and from its coupled quartet methine H-7 (δ_H_ 5.21, *J* = 6.5) to C-14 and a carbonyl C-15 (δ_C_ 172.5). These data suggested the central cyclohexene ring is part of a larger bicyclic tetrahydroisobenzofuranone system, which forms the core of the structure. A final acetoxymethyl substituent was found on the fused ring structure connected at C-13, and is supported in its assignment by a COSY correlation from H-13 (δ_H_ 2.83) to H_2_-16 (δ_H_ 4.21), as well as HMBC correlations from H_2_-16 to C-12, C-14, a carbonyl C-21 (δ_C_ 171.0) and an acetate methyl C-22 (δ_C_ 21.0). The only HMBC correlation found from H_3_-22 (δ_H_ 2.06) is to its adjacent carbonyl C-21, establishing the planar structure as **12** (Figure 5). The relative configuration of dendrillin D was supported in part by the prior analysis of dendrillin C (**11**), including the axial nature of H_3_-20 (δ_H_ 0.94) and H_3_-19 (δ_H_ 0.92), which was supported by NOESY correlations (Figure 5) of both of them to equatorial Hβ-5 (δ_H_ 1.41). NOESY correlation was also observed from equatorial H_3_-18 (δ_H_ 0.99) to axial Hα-5 (δ_H_ 1.11). These data established the trimethylcyclohexane ring as conformationally the same as **11**. The orientation of C-7 was assigned through NOESY correlations from H_3_-17 (δ_H_ 1.45) to equatorial H_3_-18, and from H-7 (δ_H_ 5.21) to equatorial Hβ-5 and axial H_3_-20, placing H_3_-17 in the α-orientation and H-7 in the β-orientation. The α-orientation of H-9 (δ_H_ 2.18) was then assigned via a NOESY correlation between H-9 and H_3_-17, as well as H-1α and H-5α, placing C-11 in the β-orientation. The final stereocenter about C-13 is established with the acetoxymethyl in the β-orientation owing to NOESY correlations from H_2_-16 (δ_H_ 4.21) to β oriented H_2_-11 (δ_H_ 1.74) and axial H_3_-20.

### 2.2. Semisynthetic Studies

**Figure 6:**
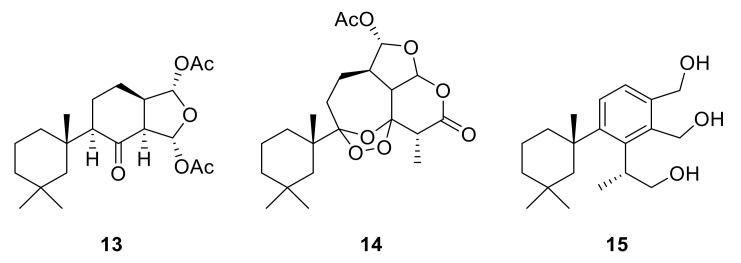
Structures of the semi-synthetic derivatives prepared from the major spongian diterpenes from *Dendrilla antarctica*. 8-Ketodihydrogracilin (**13**) and ozonide **14** were obtained from ozonolysis of 9,11-dihydrogracilin (**3**) and tetrahydroaplysulphurin-1 (**4**), respectively; Triol **15** from LiAlH_4_ reduction membranolide (**5**).

To further explore *Dendrilla antarctica* diterpene bioactivity, three of the major natural products were subjected to derivatization procedures (Figure 6). Both 9,11-dihydrogracilin A (**3**) and tetrahydroaplysulphurin-1 (**4**) were treated under reductive ozonolysis conditions. The expected 8-ketodihydrogracilin (**13**) [28] (see Figure S22) was recovered from reaction with **3**. The same reaction conditions applied to tetrahydroaplysulphurin-1 (**4**) did not lead to isolation of a di-ketone derivative owing to rapid degradation. However, a product could be purified from this reaction. 1D and 2D NMR analysis, taken with mass spectrometry, found a formula of C_22_H_32_O_8_, bearing three more oxygen atoms than the original compound. The presence of two quaternary carbons at 108.5 and 117.2 ppm, with the remaining atoms assignable to a tetrahydroaplysulphurin-like scaffold, secured the product as the ozonide (**14**) (Figures S23–27). In contrast to their traditional role as intermediates in oxidative alkene cleavage, 1,2,4-trioxolanes with bulky substituents, as found in **14**, can be relatively stable compounds. Such stabilized ozonides have found interest in treatment of malaria based on their resemblance to the natural product artemisinin [29].

The third semisynthetic product prepared for our structure-activity profile was derived by the lithium aluminum hydride (LAH) reduction of membranolide (**5**), which yielded the corresponding triol (**15**) (Figure 6). Spectroscopic analysis (Figure S30) was in agreement with that previously reported [19].

### 2.3. Biological Profiling

The full suite of *Dendrilla antarctica*-derived compounds, with the exception of darwinolide (**1**) and dendrillin D (**12**) owing to mass limitations, were tested against *Leishmania donovani* infected macrophages [15], liver-stage sporozoites in primary human hepatocytes [30], and against MRSA biofilm. Cytotoxicity was evaluated using the mammalian J774A.1 cell line in *L. donovani*-active compounds, while primary human hepatocytes were evaluated for malaria-active compounds.

The results from the *Leishmania donovani* assay (Table 2) indicate activity commensurate with the positive control and current standard of care, miltefosine (2.9 µM) is observed in aplysulphurin (**2**, 3.1 μM), tetrahydroaplysulphurin-1 (**4**, 3.5 μM), dendrillin B (**10**, 3.5 μM), 8-ketodihydrogracilin (**13**, 4.5 μM), and triol **15** (4.5 μM). 9,11–Dihydrogracilin A (**3**, 9.1 μM), membranolide (**5**, 9.7 μM), glaciolide (**6**, 8.8 μM), dendrillin A (**9**, 6.0 μM), and the semi-synthetic tetrahydroaplysulphurin ozonide (**14**, 9.9 μM) were only slightly less active. A high selectivity index (SI, mammalian cytotoxicity/antiprotozoal activity) was observed for **4** (>37), **10** (>40), and **15** (>38).

For the *Plasmodium falciparum* infected liver cells, screening was conducted at a fixed concentration of 5 µg/mL for each compound, and activities representing 60% inhibition and 100% inhibition are reported in comparison with the positive control primaquine. The results (Table 2) indicate modest activity (60% inhibition) at low micromolar concentrations for tetrahydroaplysulphurin-1 (**4**, ≤13 μM) and its ozonide derivative (**14**, ≤15 μM), dispelling any boost in activity for the ozonide. The most promising bioactivity (100% inhibition) was found for semisynthetic triol **15**, (≤16 μM). Aplysulphurin (**2**) at ≤13 μM was also active at the highest level. None of the active compounds displayed discernable cytotoxicity towards uninfected primary human hepatocytes.

The MRSA biofilm assay was carried out at concentrations of 100, 50, and 25 µg/mL for each compound, and activities representing 50% and 90% eradication are reported. The results (Table 2) show that cadlinolide C (**8**), dendrillin B (**10)**, and ozonide (**14**) achieved 90% eradication at 100 μg/mL. 8-Ketodihydrogracilin (**13**) and dendrillin C (**11**) exhibited activity at 50 μg/mL, though dendrillin C was also active at the lowest level tested (25 μg/mL), achieving 50% eradication. The most active compound in the screen was membranolide (**5**), which displayed potent activity (90% eradication) at the lowest concentration tested (25 μg/mL, 58 μM), and in fact achieved nearly 100% eradication at this level, suggesting the minimum inhibitory concentration against MRSA biofilm is actually much lower than reported here.

## 3. Conclusions

*Dendrilla antarctica* is a chemically rich sponge that continues to be a source of new chemistry and promising bioactivities. Following up on previous investigations, our large field collections of this well-studied Antarctic sponge in 2016, 2017, and 2018 have provided the resources for kg-scale dry weight bulk extractions, which resulted in a library of 11 diterpenoids, of which eight were previously described. Glaciolide (**6**) and **7** are known from northern environments [23,24], but are newly reported here from *D. antarctica*. Three new natural products have been designated dendrillins B–D (**10**–**12**). In the process, noteworthy bioactivities against infectious disease models including pathogens that cause leishmaniasis, malaria, and MRSA infections were found, revealing insights into diverse structural elements that could be meaningful in future drug development efforts.

The aromatic compounds in the library (**2**, **5**, **10**), each of which contains a variant of an isobenzofuran system with an ester at position C-6, showed activity below 10 µM against the leishmaniasis parasite. Moreover, the gain of some flexibility seems to not affect the bioactivity, but rather appears to be beneficial as it reduces the cytotoxicity, as shown for tetrahydroaplysulphurin-1 (**4**) compared with its parent compound, fully unsaturated and fairly rigid, aplysulphurin (**2**). The same observation is true for the semi-synthetic triol (**15**) compared with its parent compound membranolide (**5**); the reduction of the two ester moieties led to a modest improvement in activity (from 9.7 to 4.2 µM), but dramatically decreased the cytotoxicity. In the end, these improvements are translated into a selectivity index ratio of almost 40 for the triol compared with 8 for the natural product. Another interesting feature is revealed by comparison of **4** to cadlinolide C (**8**), differing only by an acetoxy substitution at C-16. The lack of this structural unit results in a four-fold drop of the bioactivity. Very similarly, membranolide, missing substitution at C-16 position, is less active than the structurally comparable dendrillin B (**10**), suggesting that the stereochemistry at C-15 position may not be crucial. From this perspective, the activity improvement associated with the gain of flexibility resulting in the lactone opening of membranolide to produce the triol seems to validate the importance of a substituent at C-16 position. Analysis of the top hits in each bioassay (Table 3) could represent a reasonable starting point for further structure/activity (SAR) studies. The high selectivity index (>40) of the new chemical entity dendrillin B in this regard is especially encouraging and warrants continued investigation as it showed no discernable toxicity against the human cell line and was among the most potently active, rivaling the activity seen in the miltefosine control.

Another prospective pharmacophore is revealed in the activity noted against *Plasmodium falciparum* by structurally similar compounds aplysulphurin (**2**), tetrahydroaplysulphurin-1 (**4**), and its ozonide derivative (**14**), each of which contain a fused furopyranone ring system bearing an acetate at positon C-16 and are the only compounds within the small library to sport this motif. The addition of a ozonide moieties, although known to retain antimalarial activity [30], did not significantly improve the activity. Aplysulphurin (**2**) and the semisynthetic triol (**15**) in particular showed the best activity profile, displaying 100% inhibition of the parasite cultures at low micromolar concentrations with no discernable cytotoxicity towards heathy liver cells. This could be owing to the increased rigidity of the furopyranone ring system, which in aplysulphurin is fused to a planar benzene ring and could represent another promising motif practicable for optimization. However, aplysulphurin appears bioactive in many bioassays, including some cytotoxicity assays.

Several of the *Dendrilla antarctica*-derived compounds showed promising activity in the MRSA biofilm screen, including the new natural products dendrillin B (**10**), and more potently dendrillin C (**11**), which is worth noting owing to their introduction as new chemical entities that could be further optimized, as well as the robust nature of the notoriously difficult to treat MRSA biofilm, and the lack of currently effective treatments [3]. Another interesting feature is revealed by a comparison between 9,11–dihydrogracilin A (**3**) and its ozonolysis product 8-ketodihydrogracilin (**13**). The latter displayed activity when its parent compound did not. This structural difference, namely the oxidative cleavage of the double bound leading to a truncated moiety, seems to be favorable for the bioactivity. However, the most encouraging result from this assay is the activity of the known natural product membranolide (**5**), which displayed remarkable activity even at the lowest concentrations tested. Given the low cytotoxicity against J774A.1 cells (76.8 µM) and the tendency of MRSA biofilm to form plaques on surfaces, membranolide represents another promising lead in addition to darwinolide (**1**) that could potentially be developed for topical treatment after further evaluation and structural optimization.

Overall, this investigation has highlighted the robust bioactivity profile of the cold-water sponge *D. antarctica*. Besides bioactive natural products, semi-synthetic derivatives have informed structure/activity relationships and identified strategies for further study of infectious disease drug targets.

## 4. Materials and Methods

### 4.1. General Procedures

Solvents were obtained from Fisher Scientific Co. and were HPLC grade (>99% purity) unless otherwise stated. MPLC analysis and fractionation were performed on a Teledyne-Isco CombiFlash system equipped with an evaporative light scattering detector (ELSD). HPLC analysis and fractionation were performed on an Agilent 1200 system equipped with an Agilent 1200 DAD and/or Shimadzu LC20-AT or LC10-AT system equipped with a Sedex 75 ELSD and/or a SPD-10ATvp UV-Vis detector, using analytical (Phenomenex Luna Silica (250 ×4.6 mm, 5 μm)), semi-preparative (Phenomenex Luna Silica (250 ×10 mm, 5 μm)), or preparative (Phenomenex Luna Silica (250 ×21.2 mm, 5 μm)) conditions. Analytical LC/MS was performed on a Phenomenex Kinetex C18 column (50 ×2.1 mm, 2.6 μm) on an Agilent 6230 LC/TOF-MS with electrospray ionization detection. NMR spectra were acquired in CDCl_3_ with residual solvent referenced as an internal standard (δ_H_ 7.27 ppm; δ_C_ 77.0 ppm) for ^1^H and ^13^C NMR spectra, respectively. ^1^H NMR spectra were recorded on a Varian 500 MHz or 600 MHz direct-drive instrument equipped with cold-probe detection and ^13^C NMR spectra were recorded at 125 MHz. UV absorptions were measured by an Agilent Cary 60 UV/vis spectrophotometer in CH_3_OH, while IR spectra were recorded with an Agilent Cary 630 FTIR. Optical rotations were measured using an AutoPol IV polarimeter at 589 nm utilizing a 10 mm path length cell.

### 4.2. Collection of Dendrilla antarctica

Sponge samples were collected using SCUBA at depths of 10–35 m from various sites within a 3.5 Km radius around Palmer Station, Antarctica (64.7732° S, 64.0538° W) in the austral summers of 2016, 2017, and 2018. A specimen examined by Professor Rob van Soest, then at the University of Amsterdam, was originally identified as *Dendrilla membranosa*, but recently revised to *D. antarctica* [15]. Samples were cleaned and then frozen at −70 °C, followed by lyophilization, and transported back to the University of South Florida for further processing.

### 4.3. Extraction and Isolation of Natural Products

Freeze-dried *Dendrilla antarctica* from the 2016 bulk collection (1 kg) was extracted with dichloromethane (2x), which was filtered and concentrated in vacuo. The lipophilic extract (35.2 g) was absorbed onto C18 flash columns (220 g, 40 µm) and eluted with acetonitrile, yielding two fractions. The dried material (4.5 g) of the more polar terpenoid containing fraction (fraction A) from this step was then separated by normal phase MPLC using 0–100% EtOAc in hexanes gradient over 30 min on a silica flash column (330 g, 40 µm) to afford 13 separate fractions, with one containing 9,11-dihydrogracilin A (**3**), another containing aplysulphurin (**2**), an additional fraction containing tetrahydroaplysulphurin-1 (**4**), membranolide (**5**), and the novel minor metabolite dendrillin B (**10**). The less polar non-terpenoid containing fraction (fraction B) from the solid phase extraction was discarded. Purified aplysulphurin (**2**) (450 mg) was obtained directly out of its MPLC fraction by recrystallization in MeOH, while 9,11-dihydrogracilin A (**3**) (200 mg), tetrahydroaplysulphurin-1 (**4**) (51 mg), membranolide (**5**) (125 mg), and dendrillin B (**10**) (3 mg) were each purified out of their respective MPLC fractions using normal phase HPLC conditions utilizing a 5–30% EtOAc in hexanes gradient over 25 min after 5 min at 5% EtOAc on a silica column (250 × 10.0 mm, 5 μm). Known structures were assigned based on comparison with their published data [19,21,22].

Another larger bulk collection from the 2017 field season was then extracted and fractionated using the same protocol as the previous 2016 collection, and the focus was placed on isolation of minor components. The terpenoid containing fraction (fraction A) from the solid phase extraction of this 3.34 kg of freeze-dried *D. antarctica* again yielded 13 separate MPLC fractions, with one again containing 9,11-dihydrogracilin A (~100 mg) (**3**), as well as minor constituents glaciolide (**6**) (1.7 mg), a norditerpene **7** (2.9 mg), and previously undescribed dendrillin C (**11**) (3.9 mg) purified by NP HPLC using a 0–10% EtOAc gradient in hexanes over 20 min. Aplysulphurin (**2**) was again recrystallized out of its own MPLC fraction (4g) and used for methanolysis studies. An additional MPLC fraction again contained tetrahydroaplysulphurin-1 (**4**) and membranolide (**5**); however, no additional dendrillin B (**10**) was found. One final MPLC fraction yielded cadlinolide C (**8**) (2 mg). Known structures were again assigned based on comparison with their published data [19,21,22,23,24,25].

One final round of isolations was made when additional minor constituents (**9** and **12**) were detected in several sponges collected during the 2018 field season, which were previously analyzed for a separate metabolomics study. While their masses did not contribute significantly to the metabolomic analysis, an attempt was made to isolate these components by combining the worked-up extracts of four sponges, resulting in a combined extract mass of 182.1 mg. This extract was subjected to NP analytical HPLC with a binary solvent gradient starting and holding at 5% EtOAc for the first 10 min, ramping up to 20% EtOAc over the next 10 min, increased to 100% EtOAc over the following 5 min, and finally held at 100% EtOAc for 5 min. This ultimately resulted in the isolations of new natural product dendrillin D (**12**) (0.7 mg) collected at a retention time of 20.3 min, dendrillin A (**9**) (1.8 mg) collected at a retention time of 22.0 min, as well as additional amounts of 9,11-dihydrogracilin A (**3**) (~60 mg) collected at a retention time of 13.8 min. Known structures (**3**, **9**) were assigned based on comparison with their published data [20,24].

*Dendrillin* B (**10**): clear oil; [α]^25^_D_ −34.6 (*c* = 0.26, MeCN); UV (MeOH) λ_max_ (log ε) (3.83) 245 nm; IR υ (thin film): 3372, 2940, 1738, 1718, 1385 cm^-1^; ^1^H and ^13^C NMR data, see Table 2; HRESIMS *m/z* 361.2010 [M + H]^+^ (calcd. for C_21_H_29_O_5_, 361.2010).

*Dendrillin C* (**11**): clear oil, [α]^25^_D_ −48.0 (*c* 0.10, MeCN); UV (MeOH) λ_max_ (log ε) (3.74) 230 nm; IR υ (thin film): 2937, 2781, 1740, 1696, 1670, 1387, 1381, 1184, cm^-1^; ^1^H and ^13^C NMR data, see Table 2; HRESIMS *m/z* 343.2242 [M + Na]^+^ (calcd. for C_20_H_32_NaO_3_, 343.2243).

*Dendrillin D (***12***):* clear oil; [α]^25^_D_ −40.0 (*c* = 0.10, MeCN); UV (MeCN) λ_max_ (log ε) (3.60) 220 nm; IR υ (thin film): 2940, 1748, 1736, 1387, cm^-1^; ^1^H and ^13^C NMR data, see Table 2; HRESIMS *m/z* 349.2373 [M + H]^+^ (calcd. for C_21_H_33_O_4_, 349.2373).

*Ozonolysis of tetrahydroaplysulphurin-1 (***4***):* Ozone was bubbled through a solution of tetrahydroaplysulphurin (**4**) (15 mg) in 2.5 mL of CH_2_Cl_2_ kept at 0 °C. The reaction was incubated for 5 min, after which 500 µL of dimethylsulfide was added and incubated for 45 min at 0 °C. The solvent was eliminated under nitrogen gas and residues were dissolved in 300 µL of CH_2_Cl_2_ and NP-HPLC used to isolate products. Then, 3.8 mg of ozonide (**14**) corresponding to 23% yield was isolated and used for the spectroscopic characterization. HRESIMS *m/z* 447.199 [M + Na]^+^ 447.199 calculated for C_22_H_32_O_8_Na. ^1^H NMR (CDCl_3_, 600 MHz): δ 6.29 (H-16, d, 2.2), 6.06 (H-15, d, 6.6), 3.08 (H-14, dd, 7.0, 8.0), 3.01 (H-7, q, 7.8), 2.62 (H-13, m), 2.23 (H-11α, m), 2.06 (CH_3_COO, s), 1.91-2.01 (H_2_-12, m), 1.75 (H-11β, m), 1.47 (H_2_-2, m), 1.40 (H-1β, m), 1.34 (H_3_-17, d, 7.8), 1.33 (H-3β, ov m), 1.32 (H-5β, ov m), 1.23 (H-1α, m), 1.12 (H-5α, br d, 13.6), 1.06 (H_3_-20, s), 1.01-1.05 (H-3α, m), 0.94 (H_3_-18, s), 0.87 (H_3_-19, s); ^13^C NMR (CDCl_3_, 101 MHz): δ 169.6 (CH_3_COO), 168.5 (C-6, C), 117.2 (C-9, C), 108.5 (C-8, C), 101.9 (C-15, CH), 101.8 (C-16, CH), 45.5 (C-13, CH), 43.5 (C-5, CH_2_), 43.0 (C-14, CH), 42.8 (C-7, CH), 41.4 (C-10, C), 38.7 (C-3, CH_2_), 35.5 (C-19, CH_3_), 30.6 (C-4, C), 30.5 (C-1,CH_2_), 28.7 (C-11, CH_2_), 27.5 (C-18, CH_3_), 22.6 (C-12, CH_2_), 21.1 (CH_3_COO), 20.5 (C-20, CH_3_), 18.3 (C-2, CH_2_), 11.9 (C-17, CH_3_).

### 4.4. Leishmania Donovani and J774A.1-Cell Cytotoxicity Assay

The *Leishmania donovani* cell line infected macrophage assay and cytotoxicity screen were conducted as previously described [15].

### 4.5. Liver-Stage Plasmodium Falciparum Assay

The liver-stage *Plasmodium falciparum* assay was conducted as previously reported [16,31].

### 4.6. Minimum MRSA Biofilm Eradication Concentration Assay

The MRSA biofilm assay was conducted as previously reported [17].

## Figures and Tables

**Table 1:** ^1^H and ^13^C NMR data for dendrillins B–D (**10**–**12**) ^a^.

	Dendrillin B (10)		Dendrillin C (11)		Dendrillin D (12)	
Pos.	δ_C_^b^	δ_H_^c^	δ_C_^d^	δ_H_^e^	δ_C_^b^	δ_H_^c^
1α	41.1	2.35 (1H, d, 13.6)	37.7	1.35 (1H, ov, m)	36.7	1.15 (1H, td, 12.8, 3.7)
β		1.50 (1H, m)		1.42 (1H, ov, m)		1.53 (1H, m)
2α	19.9	1.70 (1H, m)	19.6	1.54 (2H, m)	19.1	1.54 (1H, m)
β		1.79 (1H, m)				1.60 (1H, m)
3α	39.3	1.32 (2H, m)	39.1	1.11 (1H, m)	39.0	1.05 (1H, td, 12.6, 3.4)
β				1.35 (1H, ov, m)		1.44 (1H, m)
4	31.8		31.3		31.2	
5α	51.1	1.53 (1H, d, 14.3)	52.6	1.35 (1H, ov d, 13.5)	51.9	1.11 (1H, d, 13.2)
β		2.03 (1H, d, 14.3)		1.46 (1H, ov d, 13.5)		1.41 (1H, d, 12.6)
6	176.0					
7a	40.6	4.78 (1H, q, 7.3)	26.7	2.43 (1H, dq, 13.8, 7.3)	80.5	5.21 (1H, q, 6.5)
b				3.20 (1H, dq, 13.8, 7.3)		
8	136.6		167.9		169.3	
9	154.4		50.1	2.29 (1H, br t, 4.3)	47.9	2.18 (1H, t, 7.0)
10	40.1		39.5		37.7	
11α	124.0	7.78 (1H, d, 8.3)	24.5	1.40 (1H, ov m)	20.9	1.74 (2H, m)
β				2.01 (1H, dm, 14.0)		
12α	130.4	7.76 (1H, d, 8.3)	23.5	1.82 (1H, m)	23.7	1.50 (1H, m)
β				1.91 (1H, m)		1.85 (1H, m)
13	125.9		39.4	3.60 (1H, t, 7.9)	30.7	2.83 (1H, m)
14	145.5		134.2		128.0	
15	97.3	6.70 (1H, d, 9.2)	190.5	10.18 (1H, s)	172.5	
16	168.1		174.8		64.1	4.21 (2H, m)
17	14.8	1.75 (3H, d, 7.5)	16.6	1.13 (3H, br t, 7.5)	19.7	1.45 (3H, d, 6.5)
18	27.6	0.36 (3H, s)	34.6	0.98 (3H, s)	35.5	0.99 (3H, s)
19	32.8	0.93 (3H, s)	28.9	0.92 (3H, s)	27.4	0.92 (3H, s)
20	32.2	1.40 (3H, s)	24.6	1.03 (3H, s)	21.2	0.94 (3H, s)
21	52.7	3.67 (3H, s)	51.8	3.65 (3H, s)	171.0	
22					21.0	2.06 (3H, s)
OH		4.28 (1H, d, 9.2)				

^a^ CDCl_3_; ^b^ 125 MHz; ^c^ 500 MHz, ^d^ 101 MHz, ^e^ 600 MHz, ppm [integration, multplicity, *J* (Hz)]; ov—overlapping signals; m—complex multiplet.

**Table 2:** Bioactivity of *Dendrilla antarctica*-derived compounds against a variety of infection diseases.

Compound	*Leishmania donovani* IC_50_ (μM) ^a^		*Plasmodium falciparum* Inhibition ^d^ at 5 μg/mL		MRSA Biofilm Inhibition ^f^		
	Infected Macrophage ^b^	Uninfected Macrophage	Infected PHHs ^e^	Uninfected PHHs	100 μg/mL	50 μg/mL	25 μg/mL
Positive control	Miltefosine		Primaquine		None		
	2.9	>120	97.8 (5.3)	0 (2.2)	ND	ND	ND
Aplysulphurin (**2**)	3.1 ^c^	12 ^c^	100 (8.4)	7.6 (4.8)	21.1 (15.0)	29.4 (14.9)	6.5 (5.7)
9,11-Dihydrogracilin A (**3**)	9.1 ^c^	23 ^c^	0	1.1 (1.0)	12.7 (14.3)	29.1 (9.7)	0
Tetrahydroaplysulphurin-1 (**4**)	3.5 ^c^	>130 ^c^	66.7 (10.4)	3.0 (2.4)	20.8 (15.3)	31.1 (11.6)	35.0 (10.2)
Membranolide (**5**)	9.7 ^c^	77 ^c^	59.7 (19.1)	0	100.0 (0.0)	99.9 (0.1)	99.7 (0.1)
Glaciolide (**6**)	8.8	>170	0	4.5 (3.9)	32.2 (10.5)	20.8 (8.2)	20.0 (20.7)
Compound **7**	14	18	39.3 (32.7)	0.7 (1.0)	2.9 (5.2)	28.7 (15.0)	3.9 (6.1)
Cadlinolide C (**8**)	16	>160	0	0	85.4 (5.6)	35.0 (20.5)	2.6 (3.9)
Dendrillin A (**9**)	6	9	4.3 (3.8)	2.9 (0.7)	0	0	0
Dendrillin B (**10**)	3.5	>140	0	0	90.7 (1.2)	19.4 (8.4)	37.0 (12.5)
Dendrillin C (**11**)	>31	>160	1.3 (3.2)	3.4 (4.8)	99.2 (0.1)	97.8 (0.4)	84.0 (1.0)
8-Ketodihydrogracilin (**13**)	4.5	22	0	2.3 (1.9)	99.7 (0.2)	97.7 (1.1)	5.7 (9.0)
Ozonide (**14**)	9.9	17	61.7 (24.7)	18.6 (3.4)	99.9 (0.0)	40.6 (5.3)	17.7 (12.7)
Triol (**15**)	4.2	>160	81.0 (23.1)	2.2 (1.4)	9.3 (7.8)	12.3 (9.0)	2.6 (3.6)

^a^ Single point determination. ^b^ J774A.1 macrophages. ^c^ From reference [15]. ^d^ % (Standard Deviation), n = 3. ^e^ Primary human hepatocytes (PHHs). ^f^ % (Std. Dev.), n = 6. ND: not determined.

**Table 3:** Structure/activity (SAR) study of top *Dendrilla antarctica*-derived compounds against three pathogens. Green boxes illustrate promising activity, red indicates characteristics to be improved.

	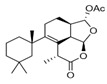	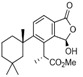	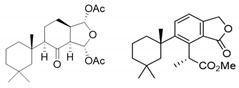		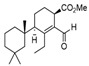	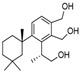
	Tetrahydro- aplysulphurum-1 (**4**)	Dendrillin B (**10**)	8-Keto- Dihydrogracilin (**13**)	Membranolide (**5**)	Dencrillin C (**11**)	Triol (**15**)
	*Leishmania* *donovani*	*Leishmania* *donovani*	*Leishmania* *donovani*	MRSA Biofilm	MRSA Biofilm	*Plasmodium* *falciparum*
Pathogen Activity	3.5 ^a^	3.5	4.5	58	78	≤16
J774 Cytotoxiciy	>130	>140	22	77	>160	>160
Selectivity Index (SI)	37	40	5	1	2.0	>10

^a^ From reference [15]; SI = J774 IC_50_/pathogen IC_50._

## Supplementary Materials

The following are available online at https://www.mdpi.com/1660-3397/18/6/327/s1, Figure S1: Dendrillin B (**10**) ^1^H NMR spectrum (500 MHz, CDCl_3_), Figure S2: Dendrillin B (**10**) ^13^C NMR spectrum (125 MHz, CDCl_3_), Figure S3: Dendrillin B (**10**) COSY spectrum (500 MHz, CDCl_3_), Figure S4: Dendrillin B (**10**) HSQC spectrum (500 MHz, CDCl_3_), Figure S5: Dendrillin B (**10**) HMBC spectrum (500 MHz, CDCl_3_), Figure S6: Dendrillin B (**10**) NOESY spectrum (600 MHz, CDCl_3_), Figure S7: Dendrillin B (**10**) HRESIMS (+), Figure S8: Dendrillin C (**11**) ^1^H NMR spectrum (500 MHz, CDCl_3_), Figure S9: Dendrillin C (**11**) ^13^C NMR spectrum (125 MHz, CDCl_3_), Figure S10: Dendrillin C (**11**) COSY spectrum (500 MHz, CDCl_3_), Figure S11: Dendrillin C (**11**) HSQC spectrum (500 MHz, CDCl_3_), Figure S12: Dendrillin C (**11**) HMBC spectrum (500 MHz, CDCl_3_), Figure S13: Dendrillin C (**11**) NOESY spectrum (600 MHz, CDCl_3_), Figure S14: Dendrillin C (**11**) HRESIMS (+), Figure S15: Dendrillin D (**12**) ^1^H NMR spectrum (500 MHz, CDCl_3_), Figure S16: Dendrillin D (**12**) ^13^C NMR spectrum (125 MHz, CDCl_3_), Figure S17: Dendrillin D (**12**) COSY spectrum (500 MHz, CDCl_3_), Figure S18: Dendrillin D (**12**) HSQC spectrum (500 MHz, CDCl_3_), Figure S19: Dendrillin D (**12**) HMBC spectrum (500 MHz, CDCl_3_), Figure S20: Dendrillin D (**12**) NOESY spectrum (600 MHz, CDCl_3_), Figure S21: Dendrillin D (**12**) HRESIMS (+), Figure S22: 8-Ketodihydrogracilin (**13**) ^1^H NMR spectrum (400 MHz, CDCl_3_), Figure S23: Ozonide **14** ^1^H NMR spectrum (600 MHz, CDCl_3_), Figure S24: Ozonide **14** ^13^C NMR spectrum (101 MHz, CDCl_3_), Figure S25: Ozonide **14** COSY spectrum (600 MHz, CDCl_3_), Figure S26: Ozonide **14** HSQC spectrum (600 MHz, CDCl_3_), Figure S27: Ozonide **14** HMBC spectrum (600 MHz, CDCl_3_), Figure S28: Dendrillin A (**9**) ^1^H NMR spectrum (400 MHz, CDCl_3_), Figure S29: Dendrillin A (**9**) ^13^C NMR spectrum (101 MHz, CDCl_3_), Figure S30: Triol **15** ^1^H NMR spectrum (400 MHz, CDCl_3_); Procedure for ozonolysis of 9,11-dihydrogracilin A (**3**) to produce 8-ketodihydrogracilin (**13**), Procedure for preparation of dendrillin A (**9**) from 9,11-dihydrogracilin (**3**), Procedure for preparation of triol **15** from membranolide (**5**).
