# Supplementary material for: Bioactivity of Spongian Diterpenoid Scaffolds from the Antarctic Sponge Dendrilla antarctica
Source: Mar Drugs. 2020 Jun 23;18(6):327. doi: 10.3390/md18060327 (PMC7344659; doi:10.3390/md18060327)
Supplement: Supplementary file 1 [file marinedrugs-18-00327-s001.pdf]

marine drugs

Supplementary Information

Bioactivity of Spongian Diterpenoid Scaffolds from the Antarctic Sponge *Dendrilla antarctica*

Alexandre Bory [1,†], Andrew J. Shilling [1,†], Jessie Allen [2], Ala Azhari [3], Alison Roth [3], Lindsey N. Shaw [2], Dennis E. Kyle [3], John H. Adams [3], Charles D. Amsler [4], James B. McClintock [4], and Bill J. Baker [1,*]

[1] Department of Chemistry, University of South Florida, 4202 E. Fowler Ave., CHE205, Tampa, Florida 33620, United States; alexandre.bory@pharma.ethz.ch (A.B.); ashillin@mail.usf.edu (A.J.S.) bjbaker@usf.edu (B.J.B.)

[2] Department of Cell Biology, Microbiology, and Molecular Biology, University of South Florida, 4202 E. Fowler Ave., ISA2015, Tampa, Florida 33620, United States; jessiea@usf.edu (J.A.); shaw@usf.edu (L.N.S)

[3] USF Center for Global Health and Infectious Diseases Research, University of South Florida, 3010 USF Banyan Circle, IDRB 304, Tampa, Florida 33612, United States; aaazhari@kau.edu.sa (A.A.); aroth1@usf.edu (A.R.); dennis.kyle@uga.edu (D.E.K.); ja2@usf.edu (J.H.A.)

[4] Department of Biology, University of Alabama at Birmingham, Birmingham, AL 35294, United States; amsler@uab.edu (C.D.A.); mcclinto@uab.edu (J.B.M.)

* Correspondence: bjbaker@usf.edu (B.J.B.); Tel.: +1-813-974-1967 (B.J.B.).

† These authors contributed equally to this paper.

Data	Page
Figure S1: Dendrillin B (**10**) ^{1}H NMR spectrum (600 MHz, $CDCl_3$).	2
Figure S2: Dendrillin B (**10**) ^{13}C NMR spectrum (125 MHz, $CDCl_3$).	3
Figure S3: Dendrillin B (**10**) COSY spectrum (600 MHz, $CDCl_3$).	4
Figure S4: Dendrillin B (**10**) HSQC spectrum (600 MHz, $CDCl_3$).	4
Figure S5: Dendrillin B (**10**) HMBC spectrum (600 MHz, $CDCl_3$).	5
Figure S6: Dendrillin B (**10**) NOESY spectrum (600 MHz, $CDCl_3$).	5
Figure S7: Dendrillin B (**10**) HRESIMS (+).	6
Figure S8: Dendrillin C (**11**) ^{1}H NMR spectrum (600 MHz, $CDCl_3$).	6
Figure S9: Dendrillin C (**11**) ^{13}C NMR spectrum (100 MHz, $CDCl_3$).	7
Figure S10: Dendrillin C (**11**) COSY spectrum (400 MHz, $CDCl_3$).	8
Figure S11: Dendrillin C (**11**) HSQC spectrum (600 MHz, $CDCl_3$).	8
Figure S12: Dendrillin C (**11**) HMBC spectrum (600 MHz, $CDCl_3$).	9
Figure S13: Dendrillin C (**11**) NOESY spectrum (600 MHz, $CDCl_3$).	9
Figure S14: Dendrillin C (**11**) HRESIMS (+).	10
Figure S15: Dendrillin D (**12**) ^{1}H NMR spectrum (500 MHz, $CDCl_3$).	10
Figure S16: Dendrillin D (**12**) ^{13}C NMR spectrum (125 MHz, $CDCl_3$).	11
Figure S17: Dendrillin D (**12**) COSY spectrum (500 MHz, $CDCl_3$).	12
Figure S18: Dendrillin D (**12**) HSQC spectrum (500 MHz, $CDCl_3$).	12
Figure S19: Dendrillin D (**12**) HMBC spectrum (500 MHz, $CDCl_3$).	13
Figure S20: Dendrillin D (**12**) 1D NOESY spectrum (600 MHz, $CDCl_3$).	13
Figure S21: Dendrillin D (**12**) HRESIMS (+).	14
Procedure for ozonolysis of 9,11-dihydrogracilin A (3) to produce 8-ketodihydrogracilin (**13**).	15
Figure S22: 8-Ketodihydrogracilin (**13**) ^{1}H NMR spectrum (400 MHz, $CDCl_3$).	15
Figure S23: Ozonide **14** ^{1}H NMR spectrum (600 MHz, $CDCl_3$).	16
Figure S24: Ozonide **14** ^{13}C NMR spectrum (101 MHz, $CDCl_3$).	16
Figure S25: Ozonide **14** COSY spectrum (600 MHz, $CDCl_3$).	17
Figure S26: Ozonide **14** HSQC spectrum (600 MHz, $CDCl_3$).	17
Figure S27: Ozonide **14** HMBC spectrum (600 MHz, $CDCl_3$).	17
Procedure for preparation of dendrillin A (**9**) from 9,11-dihydrogracilin (**3**).	18
Figure S28: Dendrillin A (**9**) ^{1}H NMR spectrum (400 MHz, $CDCl_3$).	18
Figure S29: Dendrillin A (**9**) ^{13}C NMR spectrum (101 MHz, $CDCl_3$).	18
Procedure for preparation of triol **15** from membranolide (**5**).	19
Figure S30: Triol **15** ^{1}H NMR spectrum (400 MHz, $CDCl_3$).	19

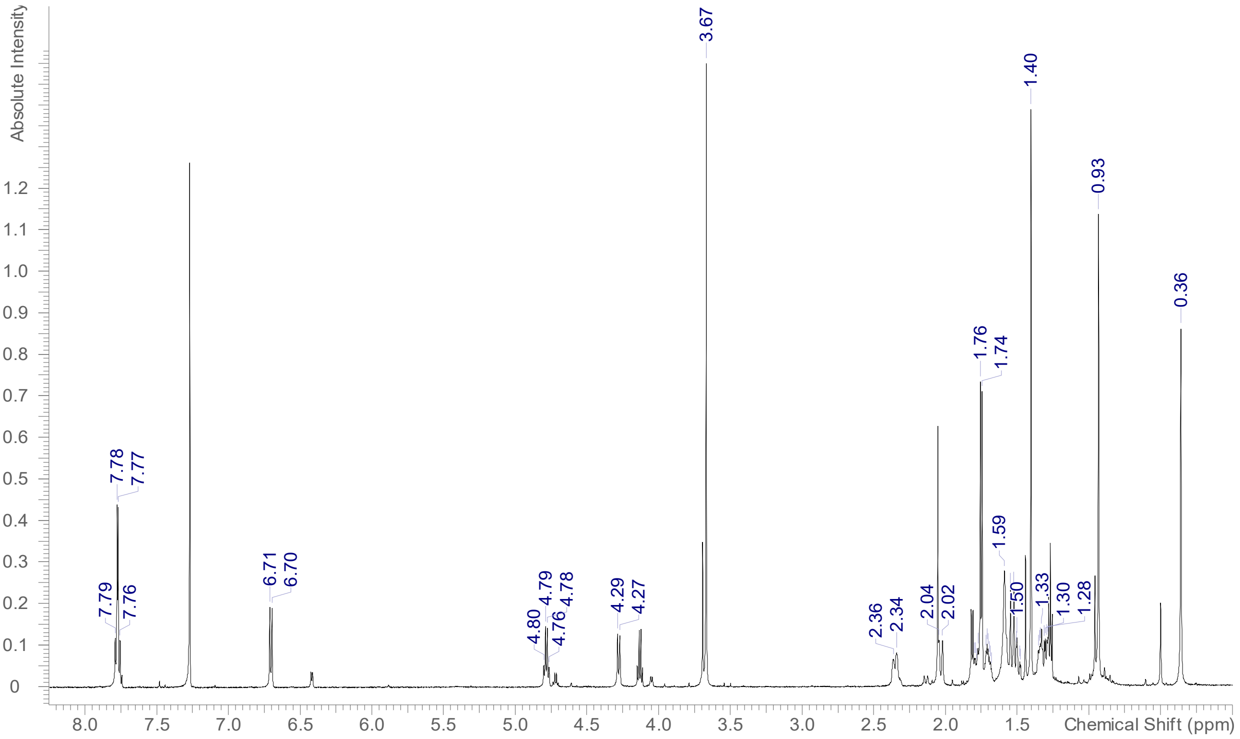

Figure S1. Dendrillin B (**10**) ^{1}H NMR spectrum (600 MHz, $CDCl_3$)

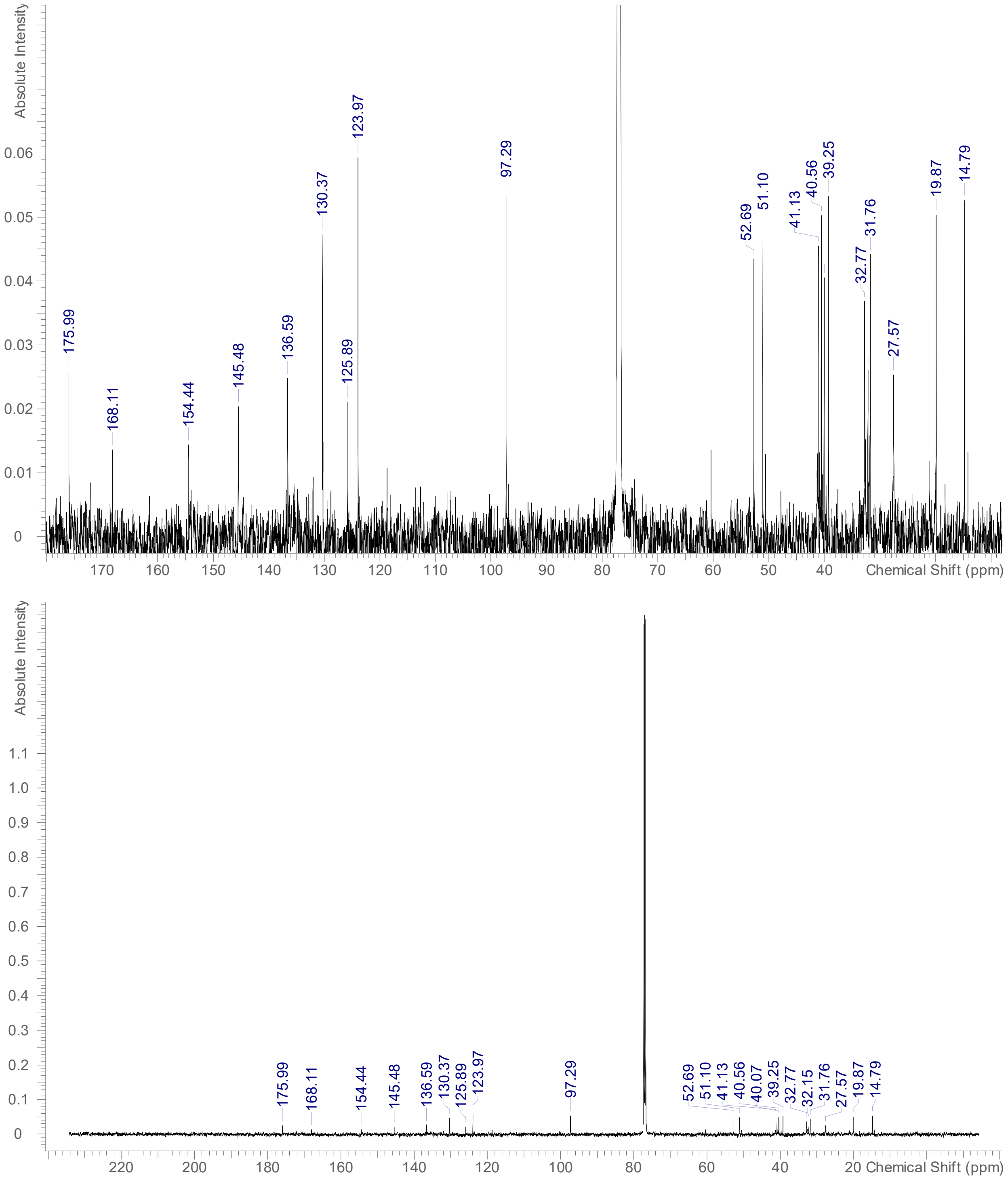

Figure S2. Dendrillin B (**10**) ^{13}C NMR spectrum (125 MHz, $CDCl_3$) (top image zoomed).

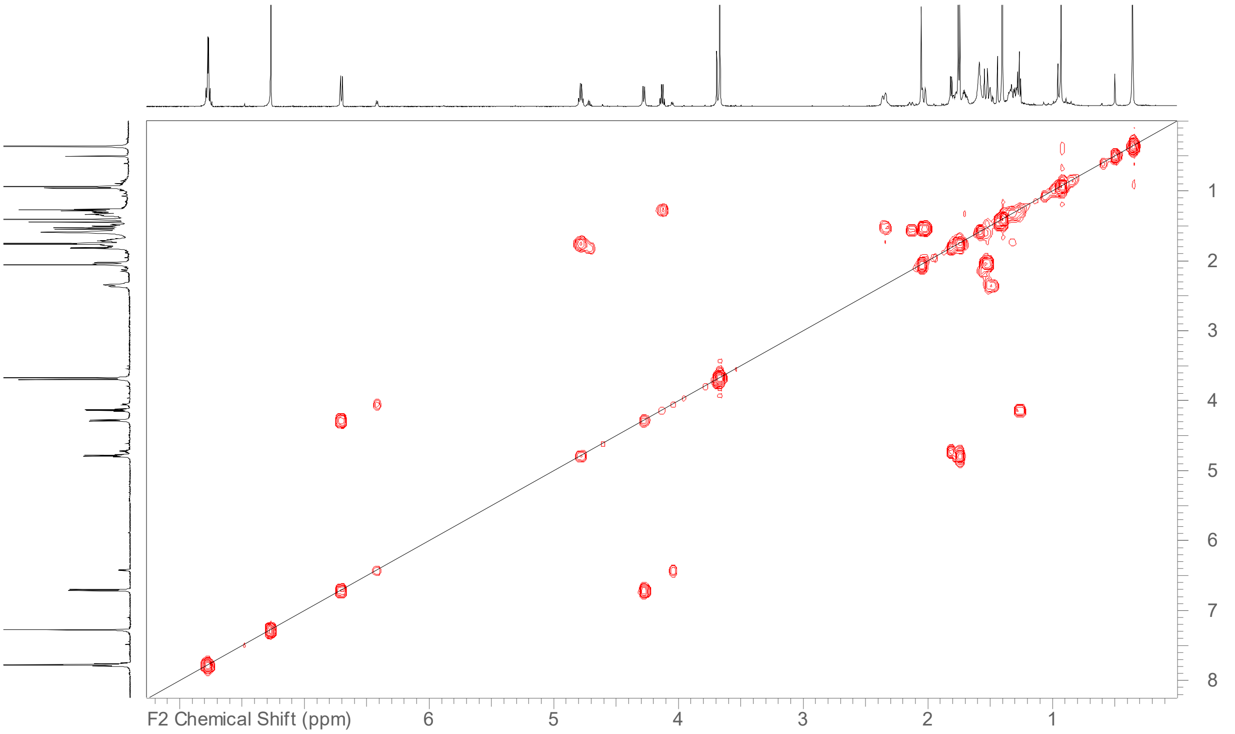

Figure S3. Dendrillin B (**10**) COSY spectrum (600 MHz, $CDCl_3$).

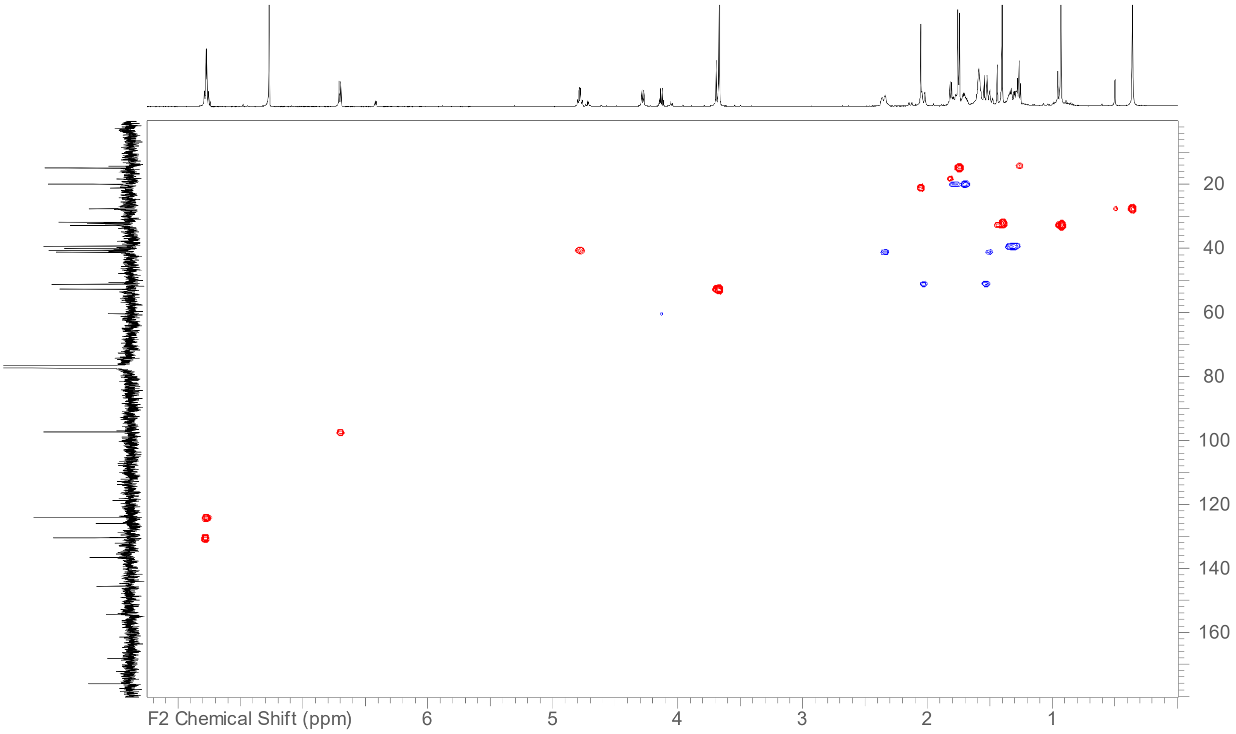

Figure S4. Dendrillin B (**10**) HSQC spectrum (600 MHz, $CDCl_3$).

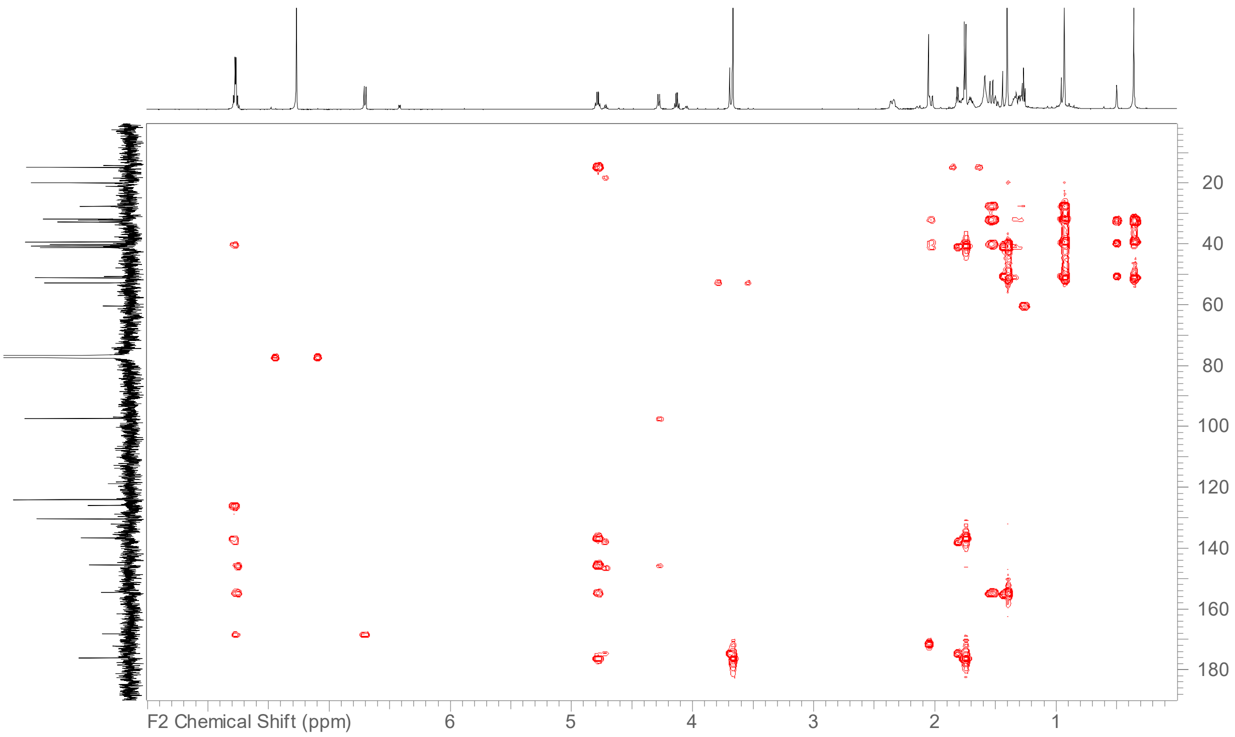

Figure S5. Dendrillin B (**10**) HMBC spectrum (600 MHz, $CDCl_3$).

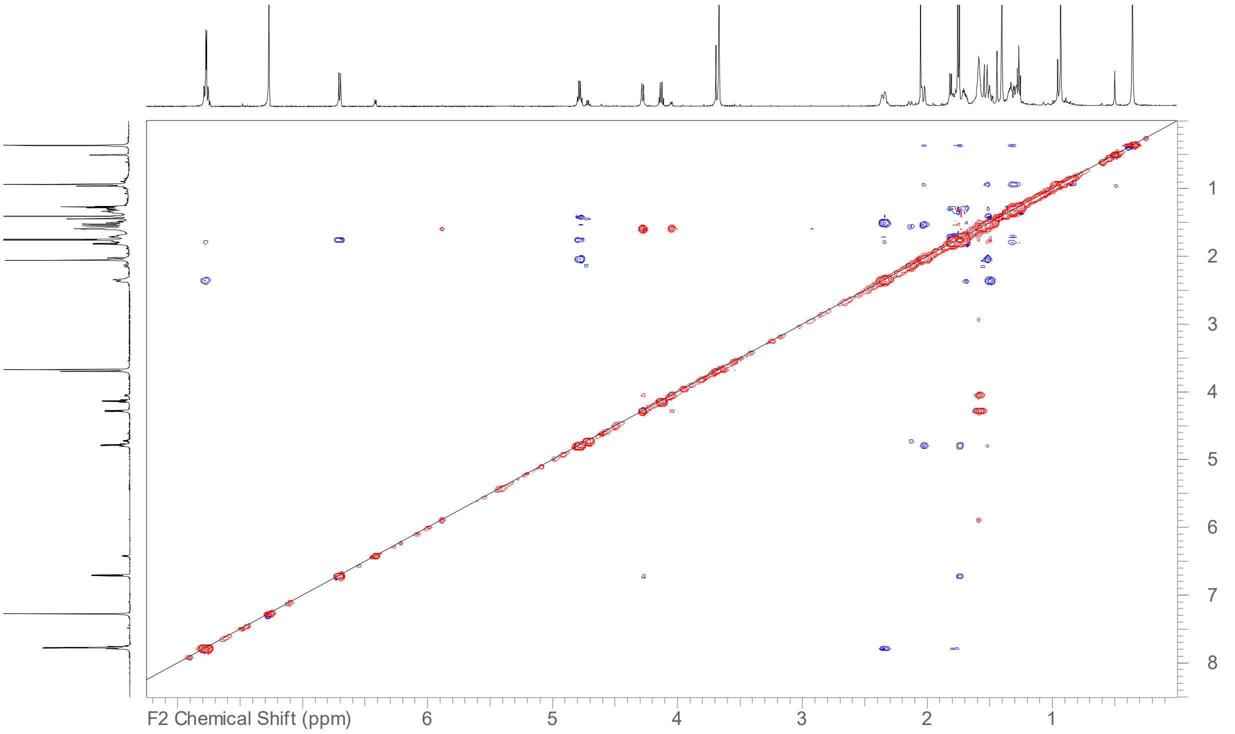

Figure S6. Dendrillin B (**10**) NOESY spectrum (500 MHz, $CDCl_3$).

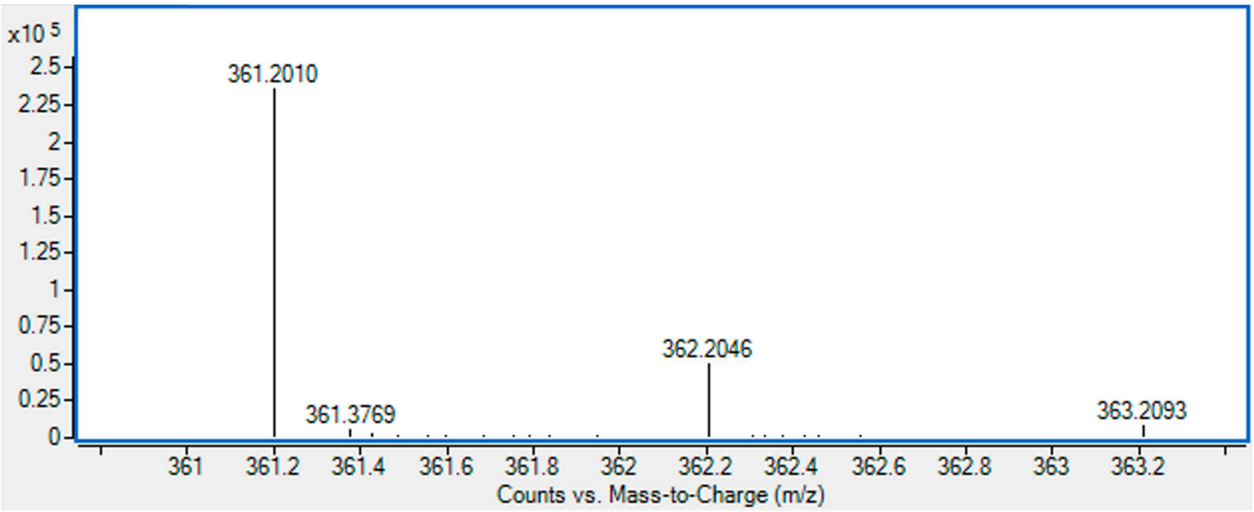

Formula	Species	Abundance (counts)	Ion Mass	Measured Mass	Error (ppm)	Error (mDa)
C21H28O5	[M+H]+	235855.88	361.2010	361.2010	0.000	0.0

Figure S7. Dendrillin B (**10**) HRESIMS (+)

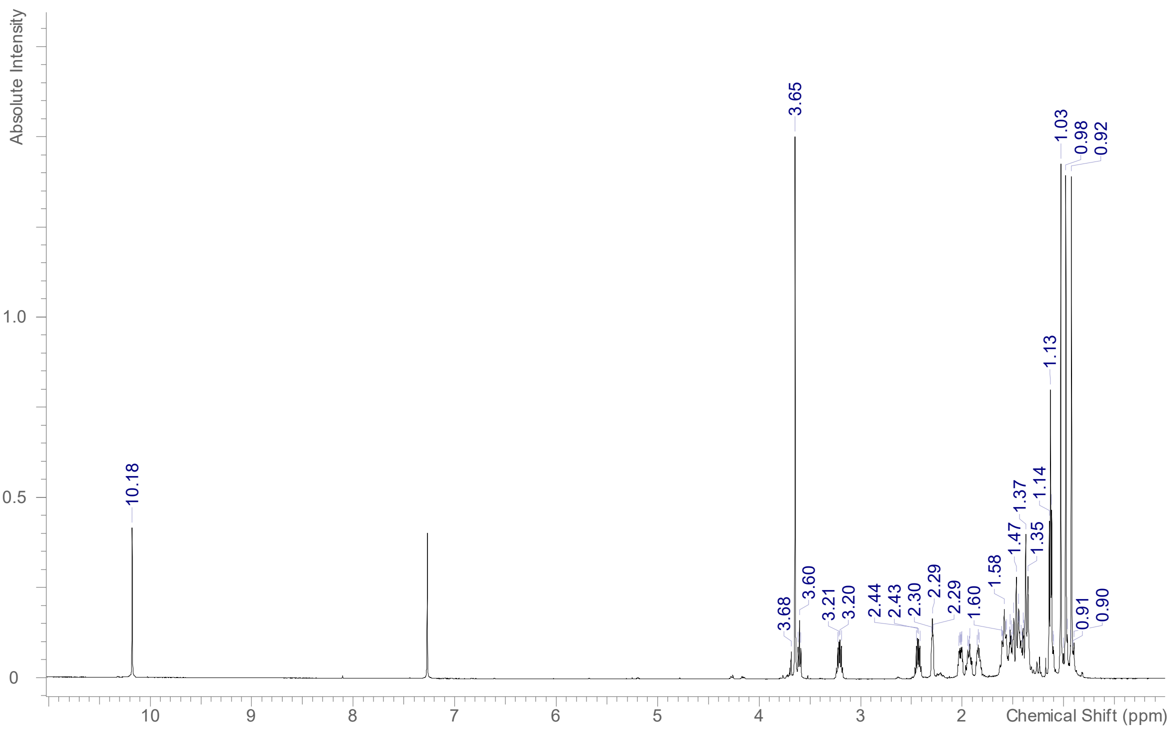

Figure S8. Dendrillin C (**11**) ^{1}H NMR spectrum (600 MHz, $CDCl_3$)

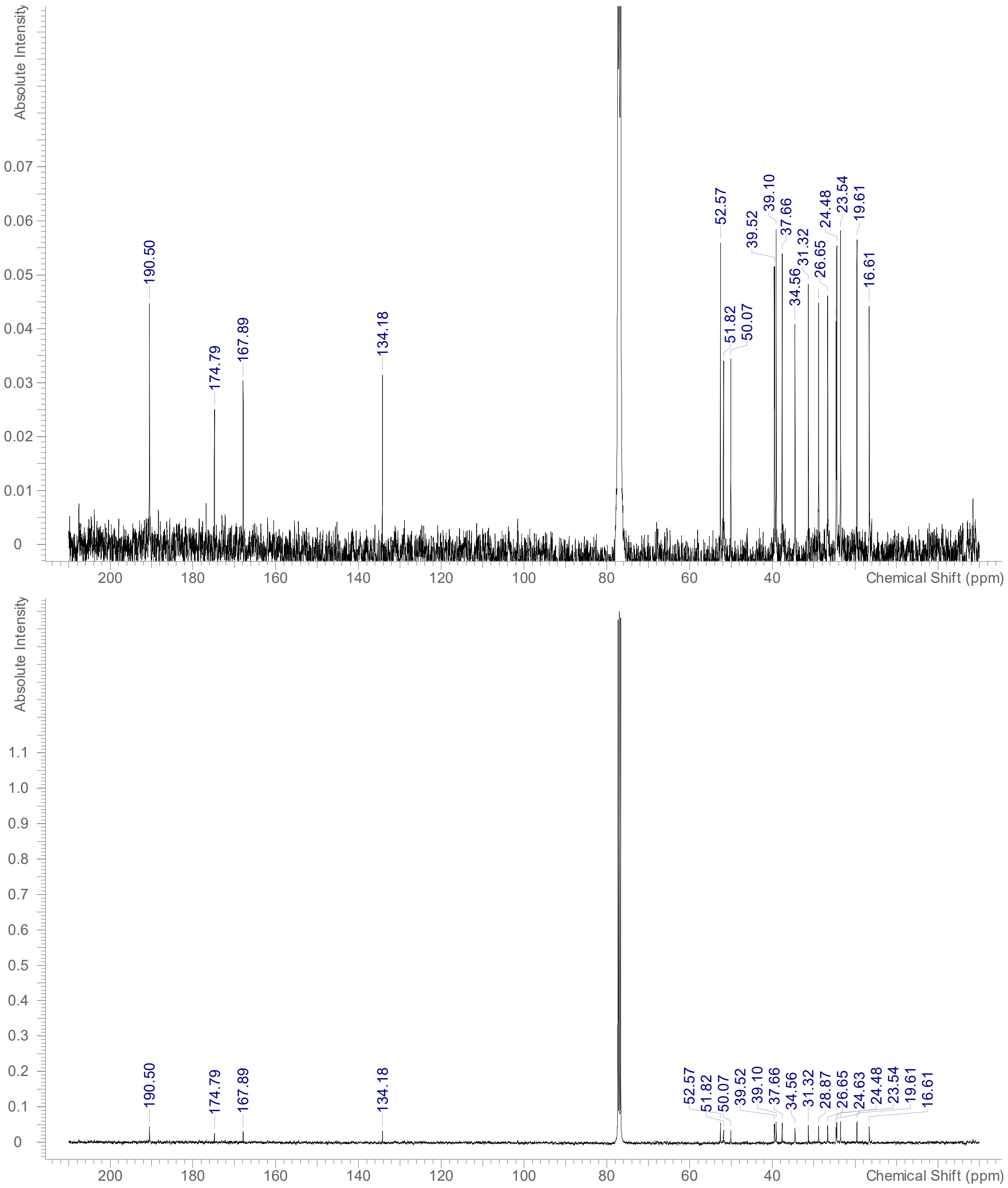

Figure S9. Dendrillin C (**11**) ^{13}C NMR spectrum (100 MHz, $CDCl_3$) (top image zoomed).

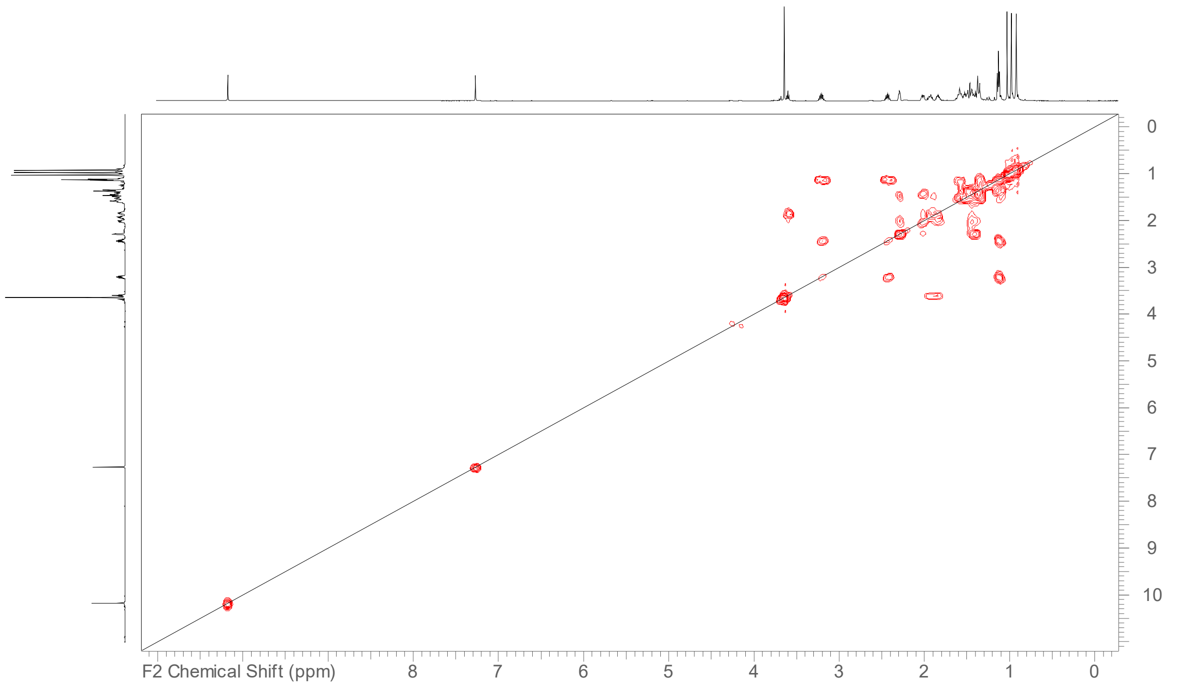

Figure S10. Dendrillin C (**11**) COSY spectrum (400 MHz, $CDCl_3$).

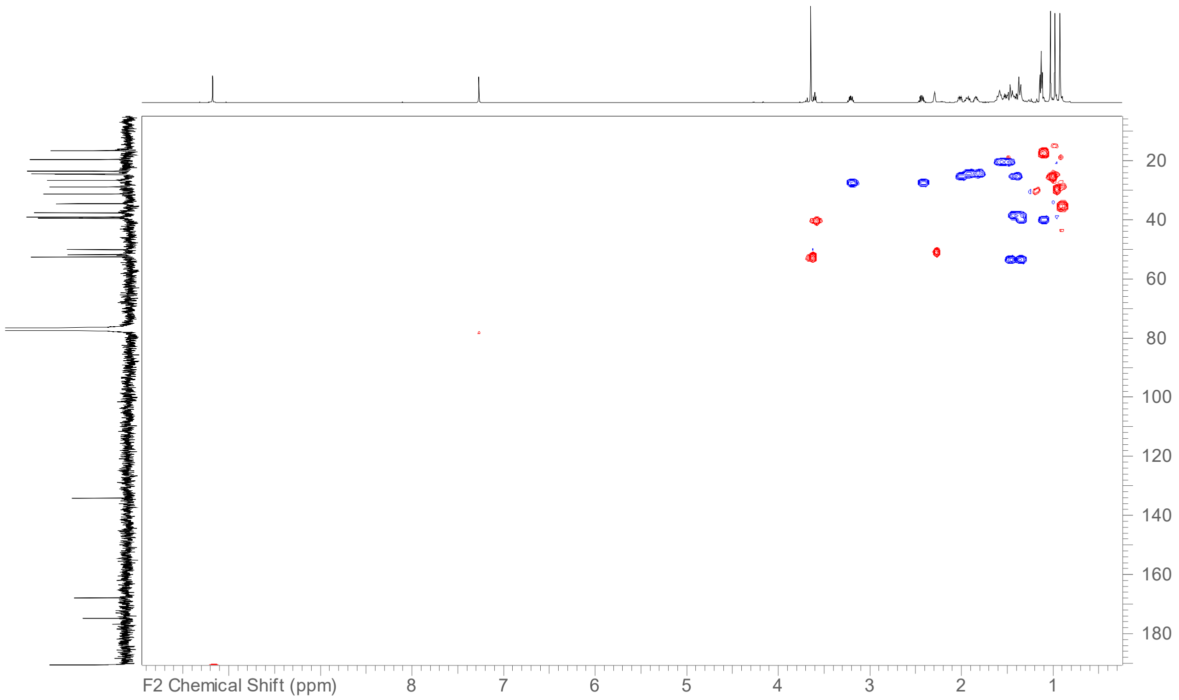

Figure S11. Dendrillin C (**11**) HSQC spectrum (600 MHz, $CDCl_3$).

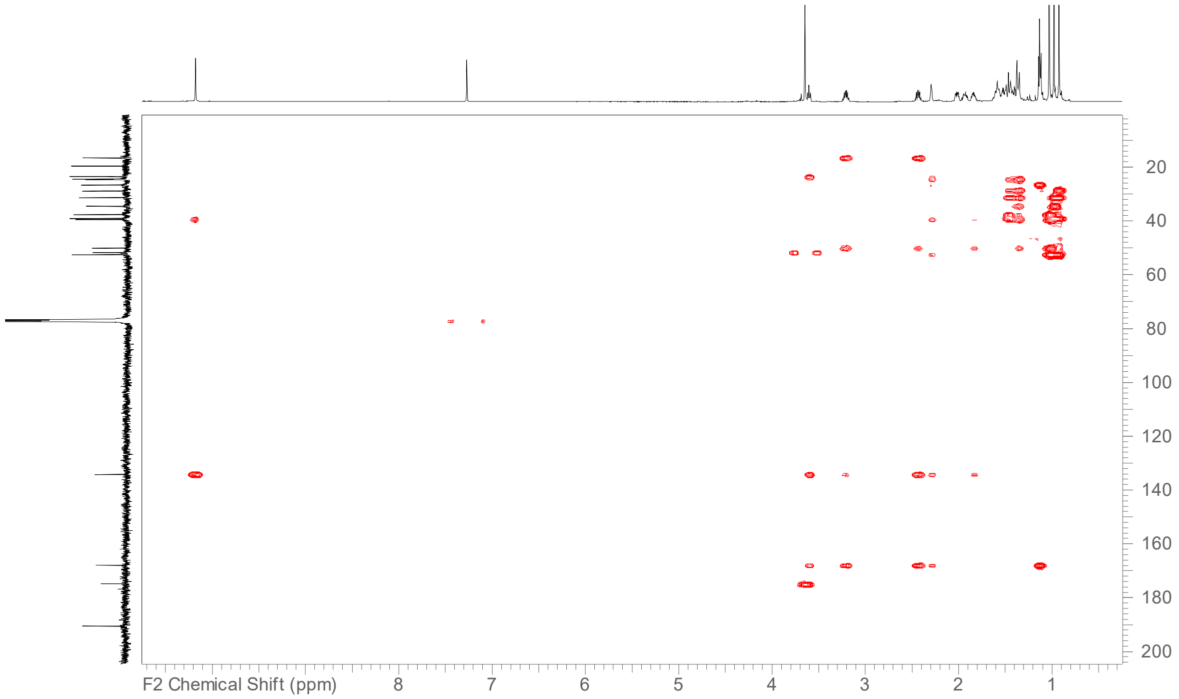

Figure S12. Dendrillin C (**11**) HMBC spectrum (600 MHz, $CDCl_3$).

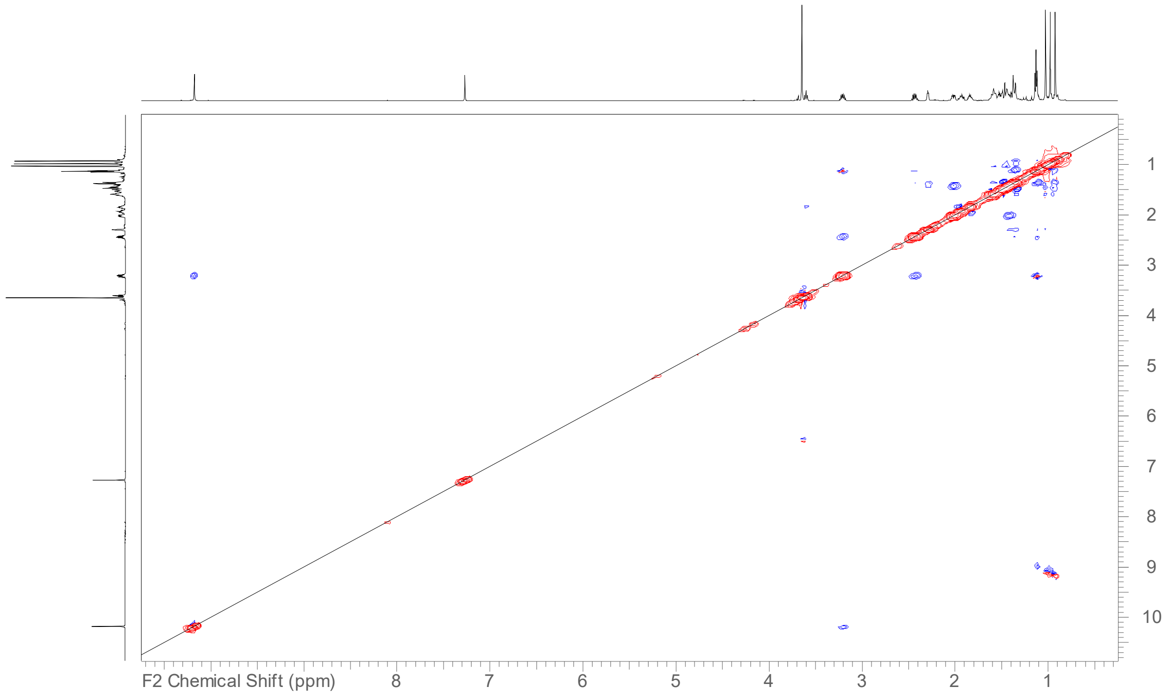

Figure S13. Dendrillin C (**11**) NOESY spectrum (600 MHz, $CDCl_3$).

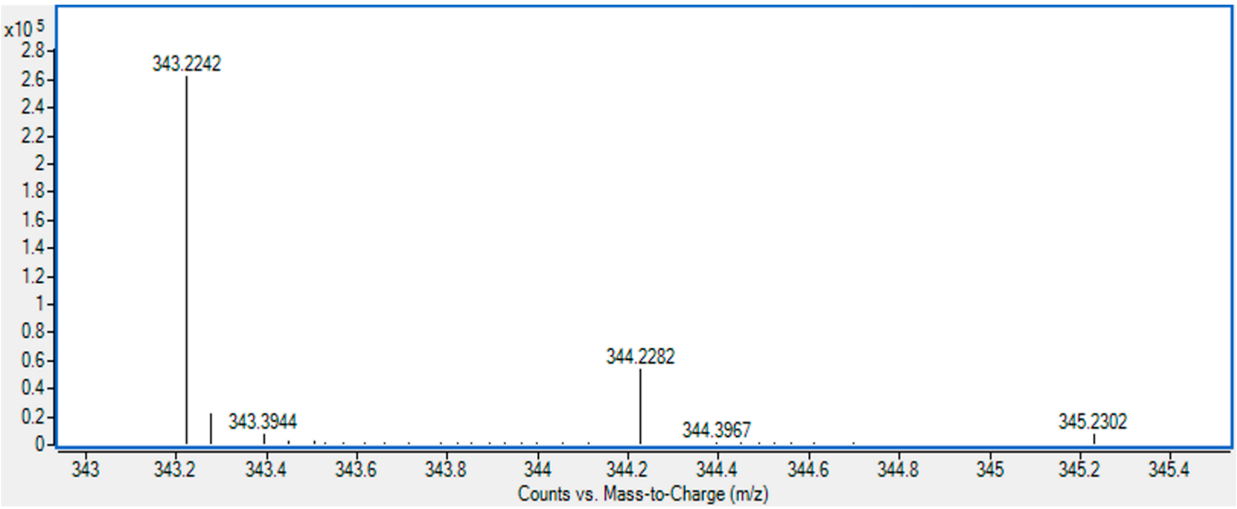

Formula	Species	Abundance (counts)	Ion Mass	Measured Mass	Error (ppm)	Error (mDa)
C20H32O3	[M+Na]+	253870.96	343.2243	343.2242	-0.291	-0.1

Figure S14. Dendrillin C (**11**) HRESIMS (+)

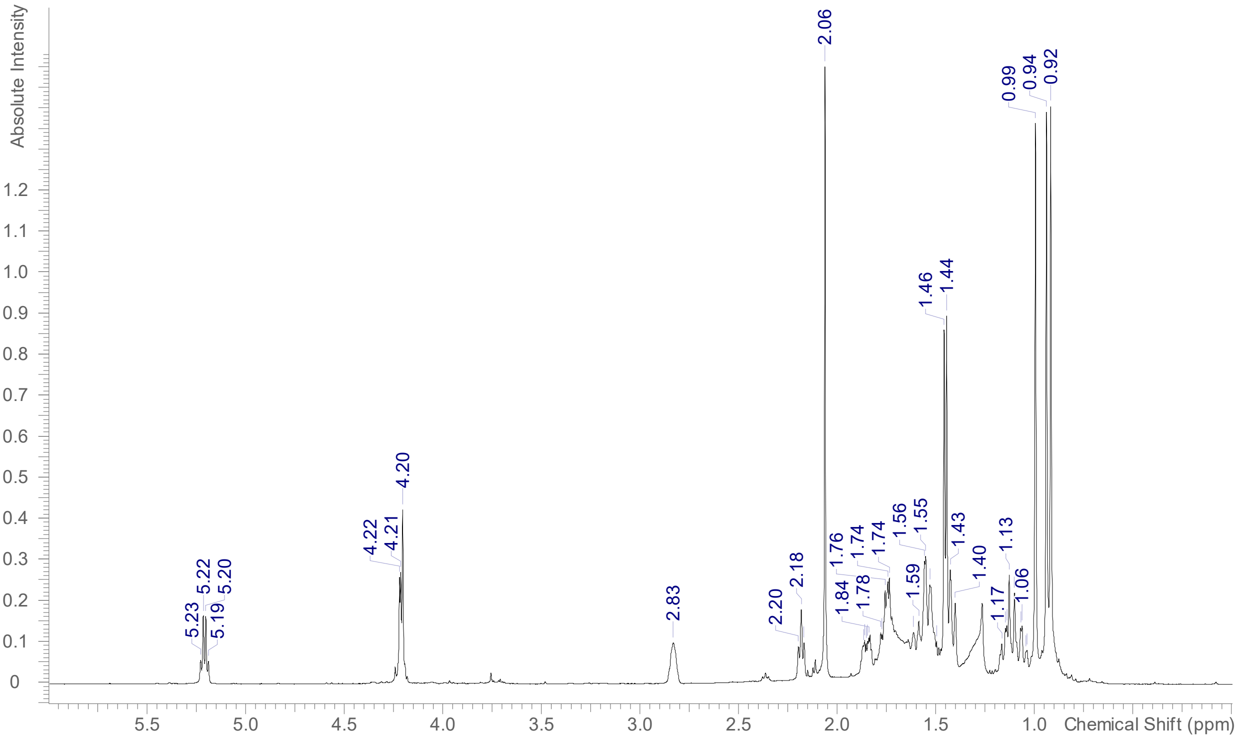

Figure S15. Dendrillin D (**12**) ^{1}H NMR spectrum (500 MHz, $CDCl_3$).

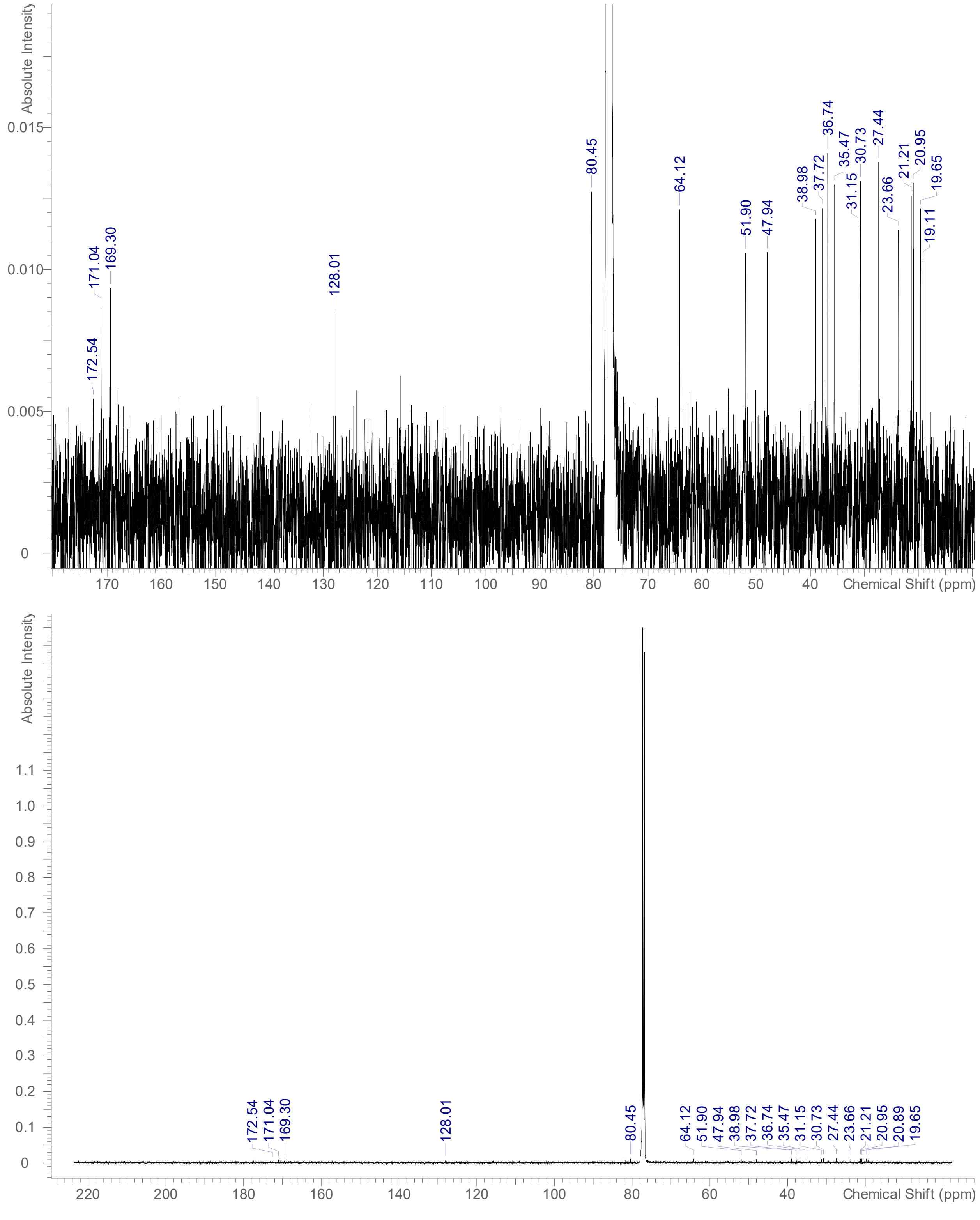

Figure S16. Dendrillin D (**12**) ^{13}C NMR spectrum (125 MHz, $CDCl_3$) (top image zoomed).

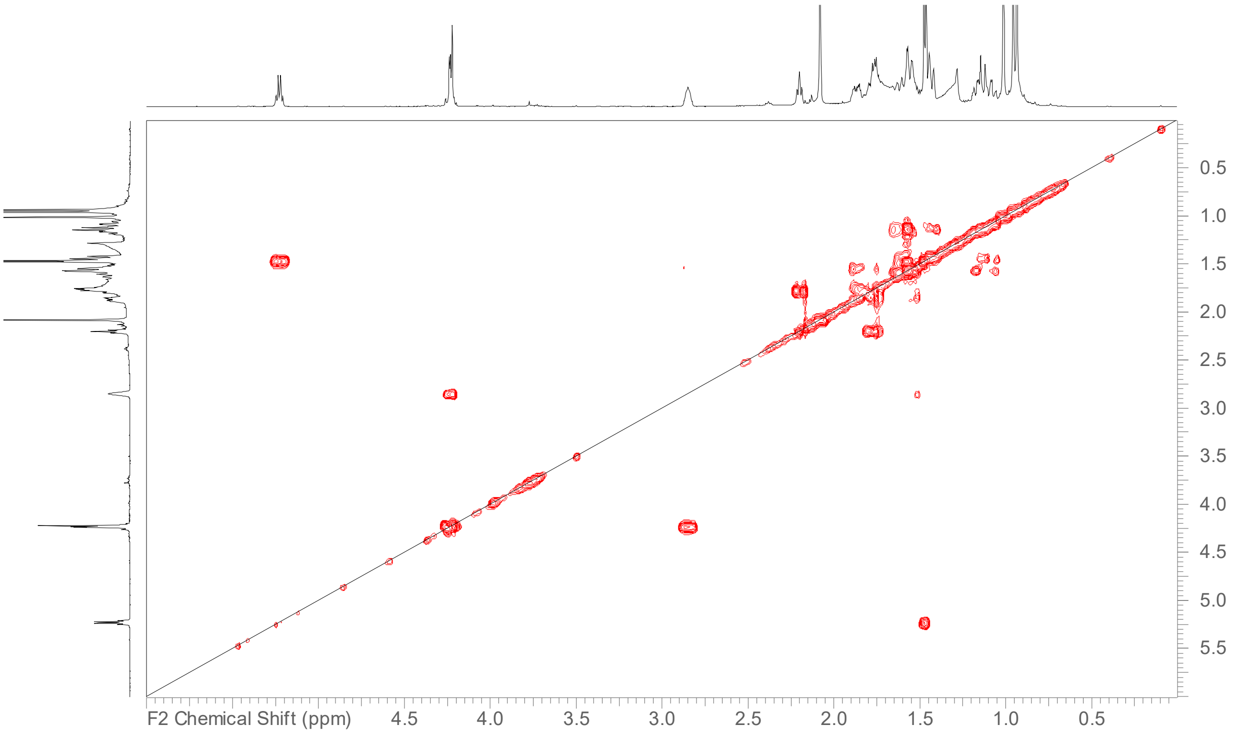

Figure S17. Dendrillin D (**12**) COSY spectrum (500 MHz, $CDCl_3$).

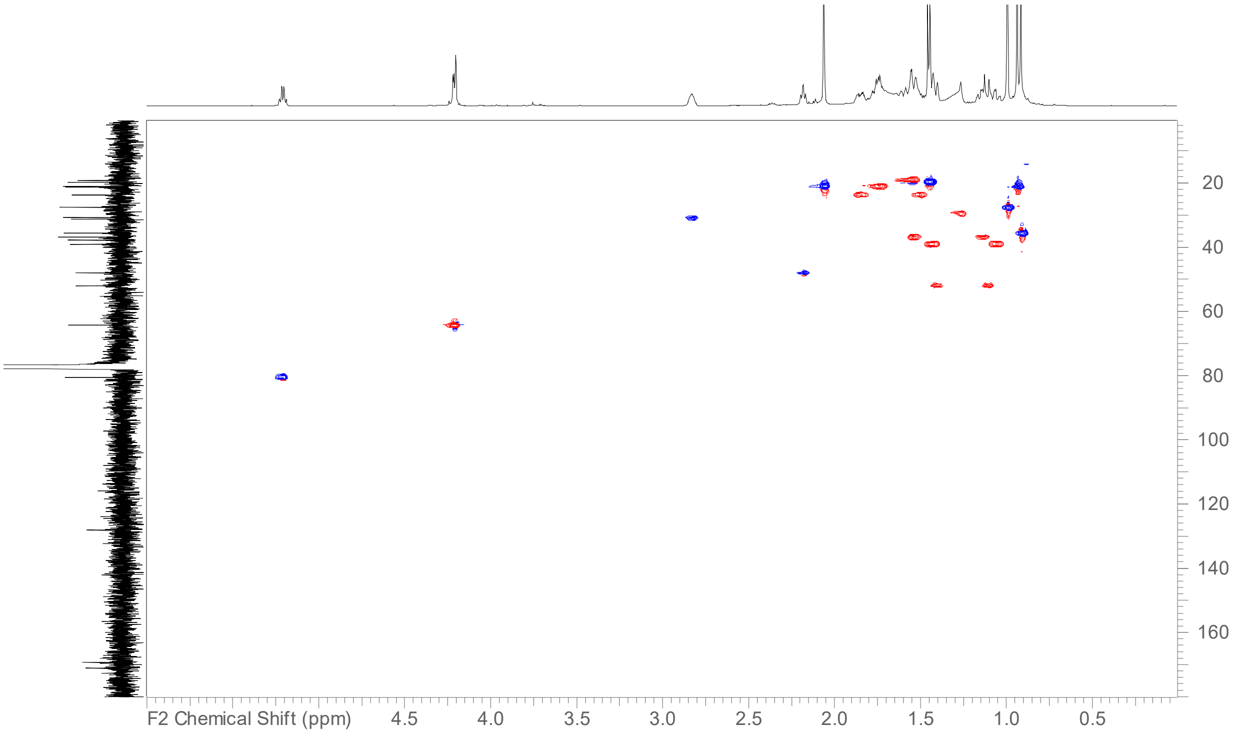

Figure S18. Dendrillin D (**12**) HSQC spectrum (500 MHz, $CDCl_3$).

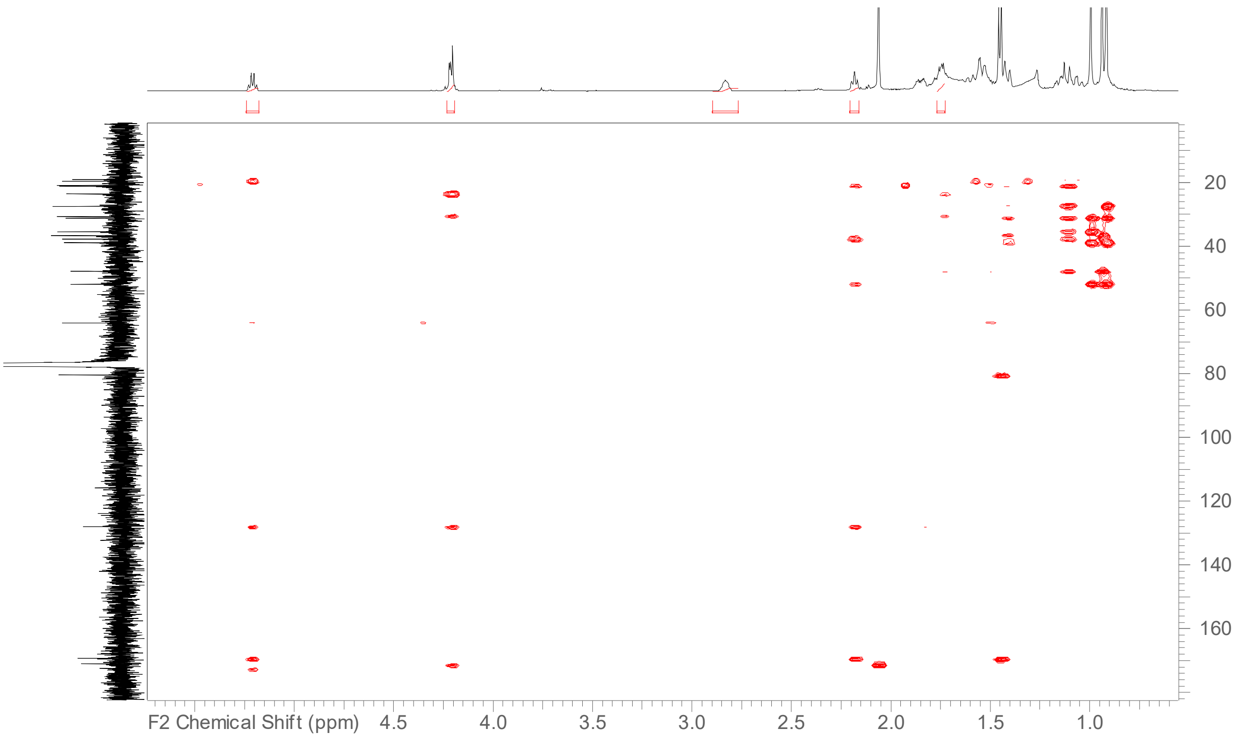

Figure S19. Dendrillin D (**12**) HMBC spectrum (500 MHz, $CDCl_3$).

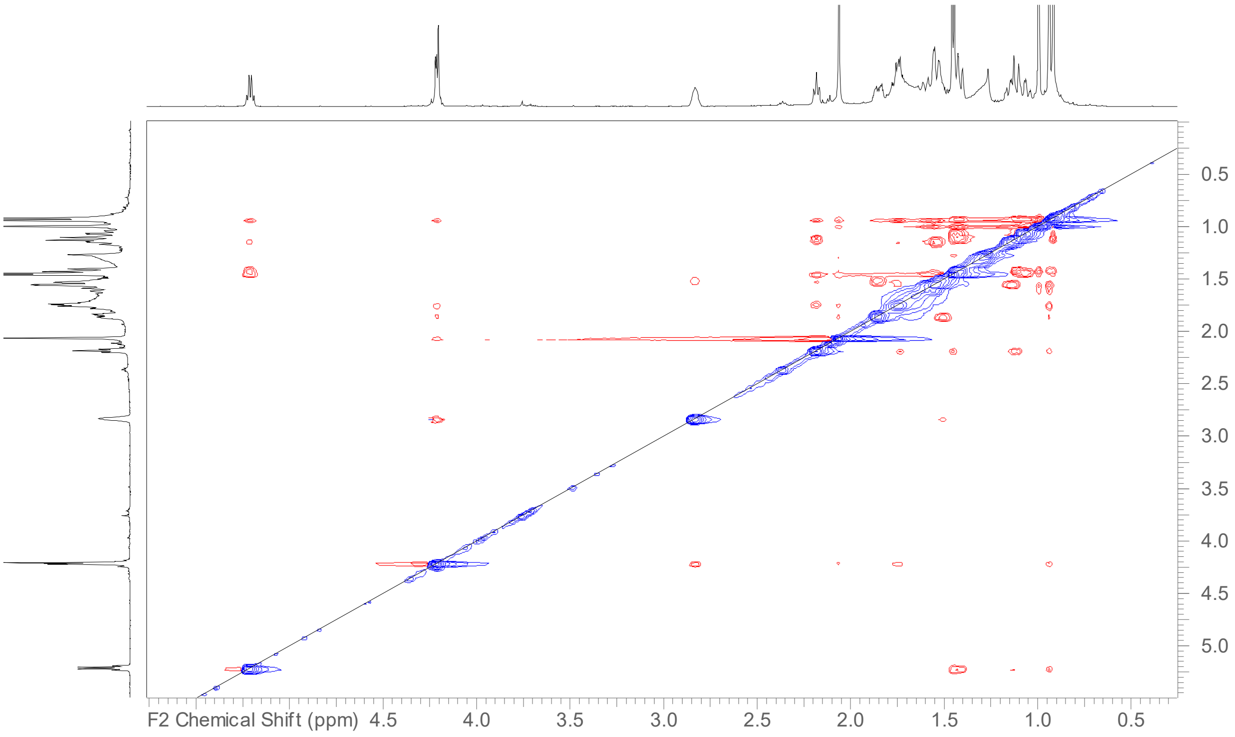

Figure S20. Dendrillin D (**12**) NOESY spectrum (500 MHz, $CDCl_3$).

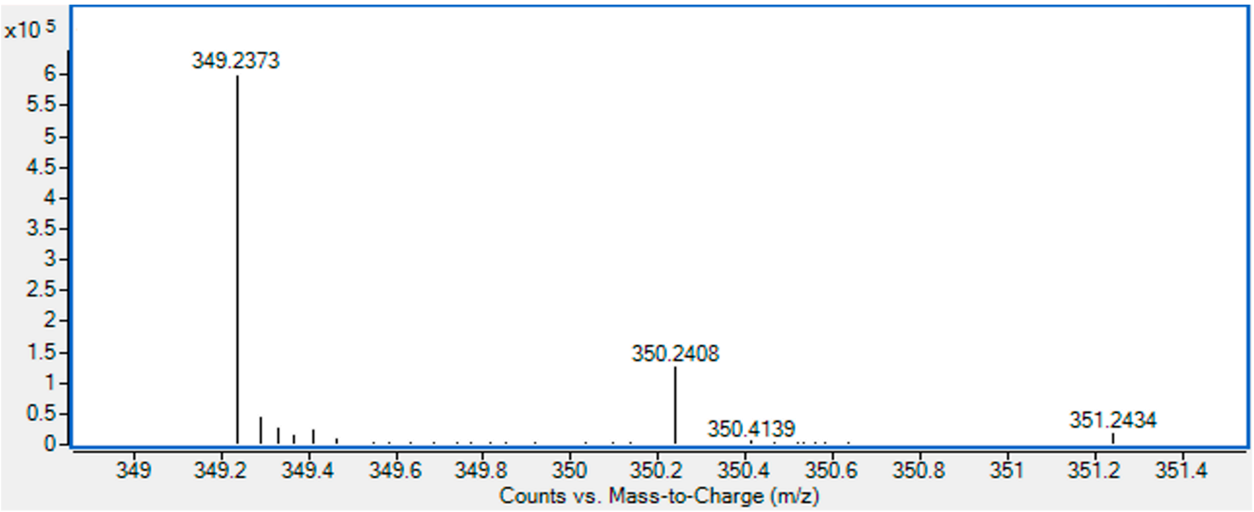

Formula	Species	Abundance (counts)	Ion Mass	Measured Mass	Error (ppm)	Error (mDa)
C21H32O4	[M+H]+	598864.50	349.2373	349.2373	0.000	0.0

Figure S21. Dendrillin D (**12**) HRESIMS (+)

*Procedure for ozonolysis of 9,11-dihydrogracilin A (**3**) to produce 8-ketodihydrogracilin (**13**):* 9,11-Dihydrogracilin A (**3**) (9 mg) was dissolved in 2.0 mL of hexanes and ozone, which was generated by O_2 flowing at 0.5 mL/min over a Telsa coil, was bubbled through the solution kept at 0 °C. The reaction was incubated for 5 min, after which 500 μL of dimethylsulfide was added and incubated for 45 min at 0 °C. The solvent was removed with nitrogen gas and residues were re-suspended in 300 μL of CH_2Cl_2. NP-HPLC was used to purify the extract and 3.5 mg (39%) of the keto-derivative (**13**) was obtained. HRESIMS *m/z* 403.209 $[M + Na]^+$ 403.209 calculated for $C_{21}H_{32}O_6Na$. ^1H NMR (400 MHz, $CDCl_3$): 1.16 (2H, m), 1.29 (2H, m, ov) 1.43 (1H, m), 1.53 (1H, m), 1.63 (1H, m), 1.78 (1H, m), 1.96 (1H, m), 2.24 (1H, dd, 11.8, 5.6). Adapted from procedure reported in: Puliti, R.; Fontana, A.; Cimino, G.; Mattia, C.A.; Mazzarella, L. Structure of a keto derivative of 9,11-dihydrogracilin A. *Acta Crystallogr., Sect. C: Crys. Struct. Commun.* **1993**, *C49*, 1373-1376.

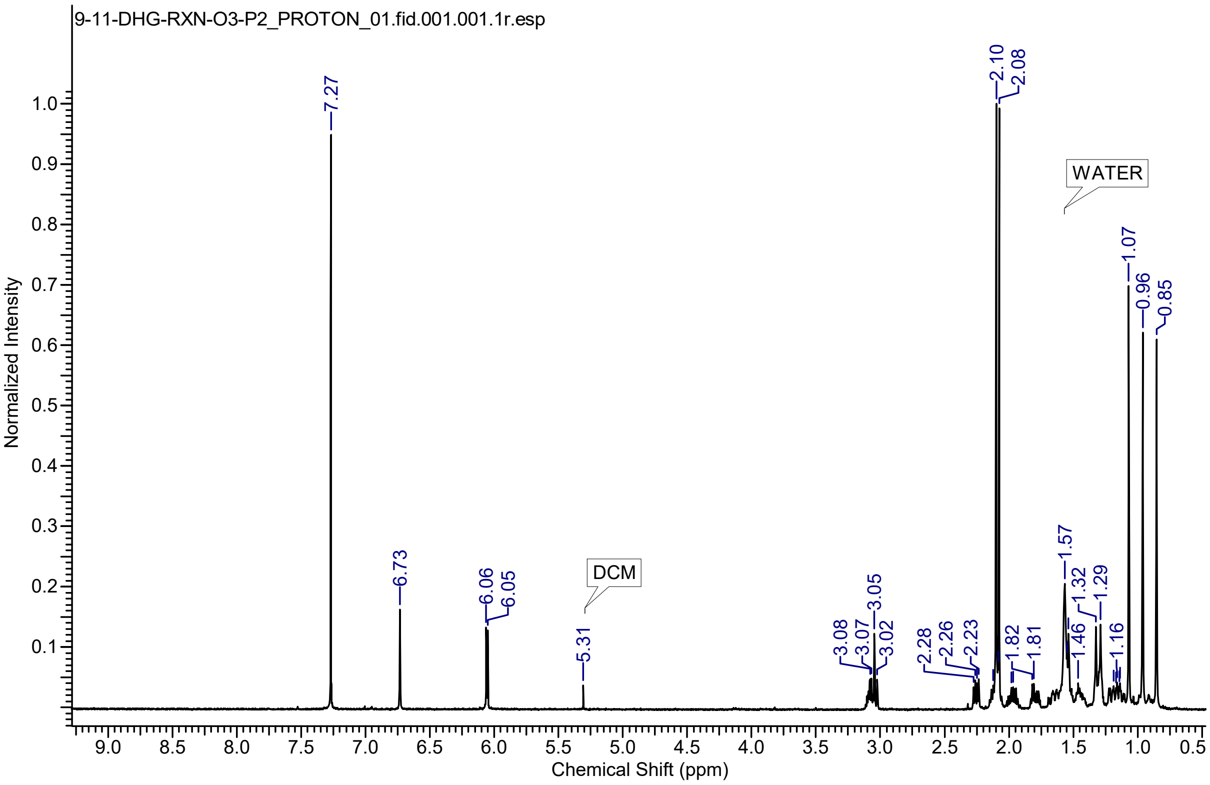

Figure S22: 8-Ketodihydrogracilin (**13**) ^1H NMR spectrum (400 MHz, $CDCl_3$).

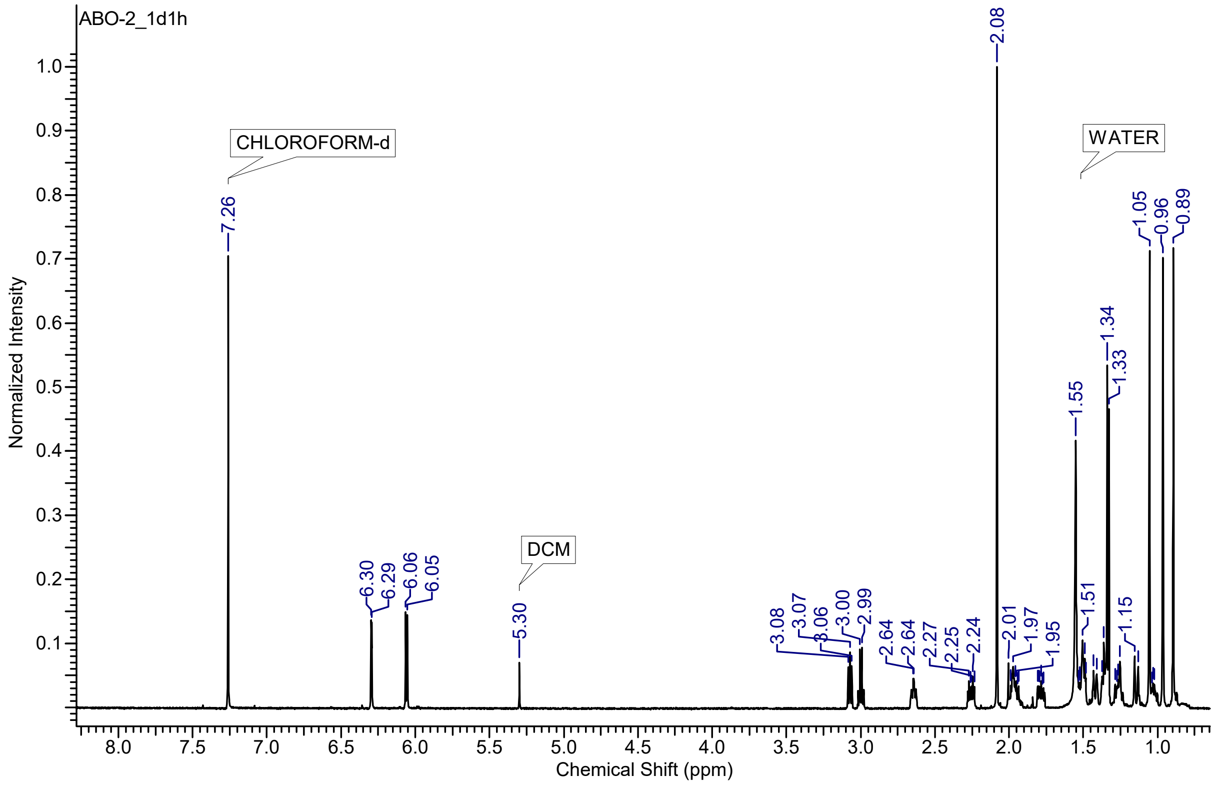

Figure S23: Ozonide **14** ^{1}H NMR spectrum (600 MHz, $CDCl_3$).

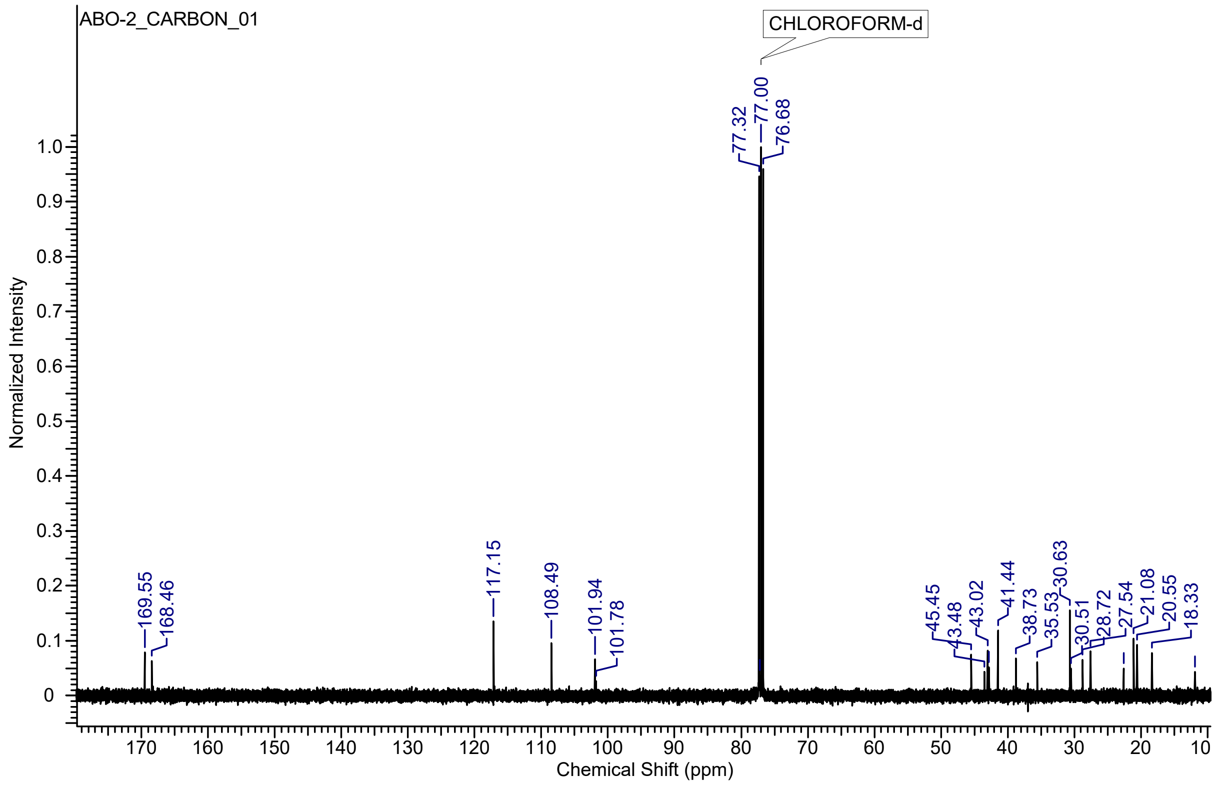

Figure S24: Ozonide **14** ^{13}C NMR spectrum (101 MHz, $CDCl_3$).

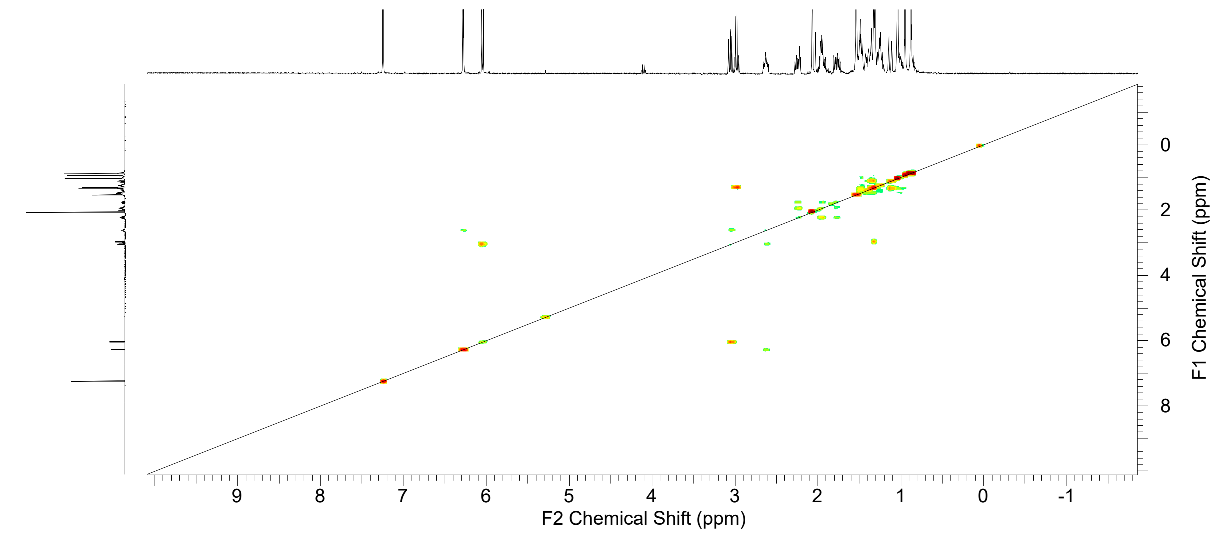

Figure S25: Ozonide **14** COSY spectrum (400 MHz, $CDCl_3$).

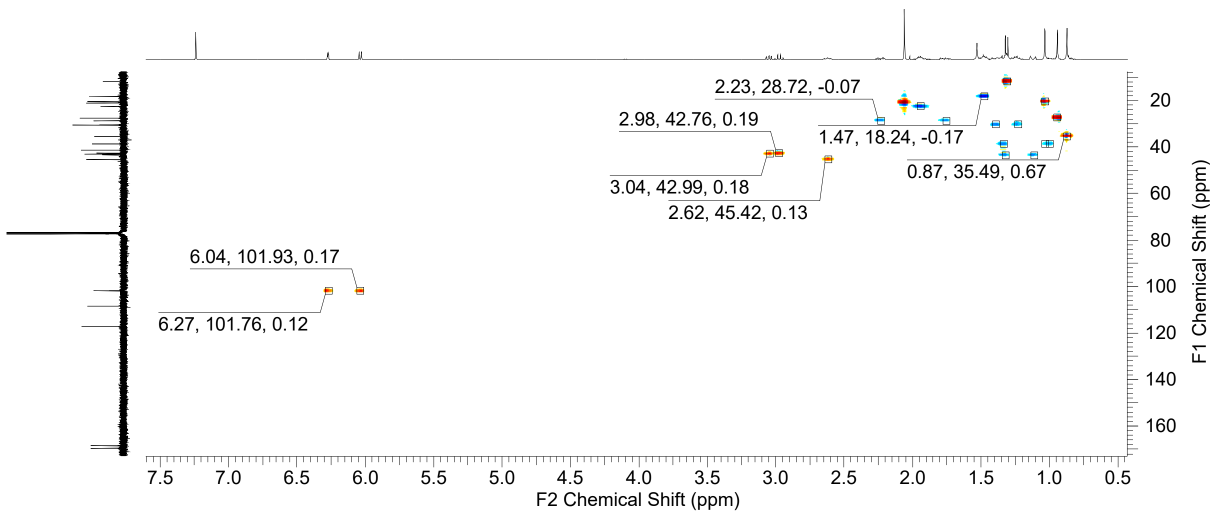

Figure S26: Ozonide **14** HSQC spectrum (400 MHz, $CDCl_3$).

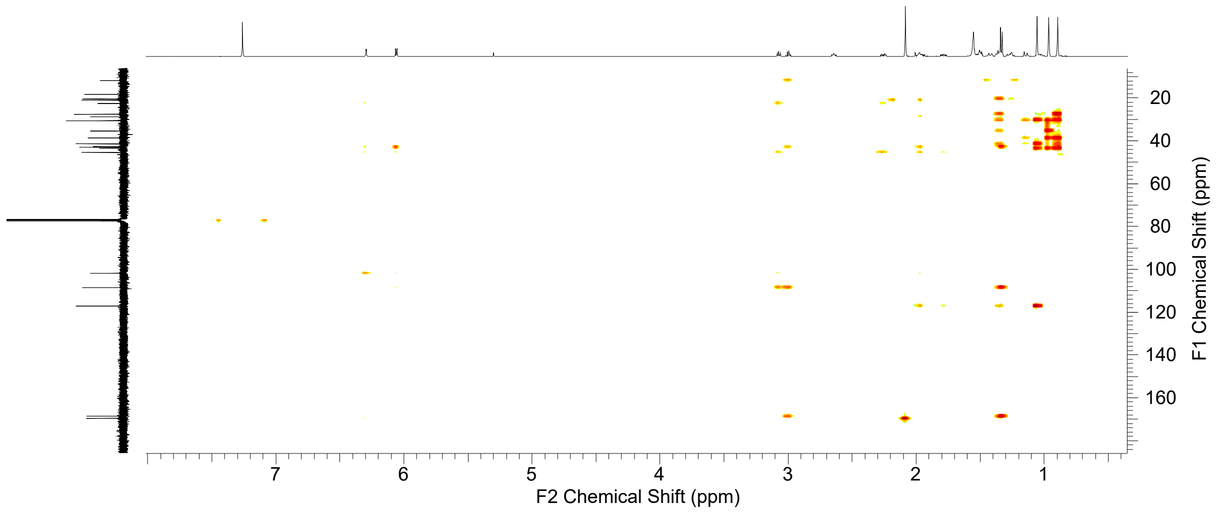

Figure S27: Ozonide **14** HMBC spectrum (400 MHz, $CDCl_3$).

Procedure for conversion of 9,11-dihydrogracilin A (**3**) *to dendrillin A* (**9**): To a solution of 47.2 mg 9,11-dihydrogracilin A (**3**) (0.12 mmol) in 10 mL of CH_2Cl_2, was added 24.2 mg of *m*-CPBA (0.14 mmol). After reflux overnight, the reaction was quenched with 20 mL of 10% NaOH (aq) and the aqueous layer re-extracted with CH_2Cl_2. Combined organic layers were purified by NP-HPLC (hexane to EtOAc) to yield 13 mg (0.03 mmol, 25%) of dendrillin A (**9**). HRESIMS m/z 431.241 $[M + Na]^+$ 431.240 calculated for $C_{23}H_{36}O_6Na$. ^1H NMR and ^{13}C NMR data in agreement with published values: Baker, B.J.; Kopitzke, R.W.; Yoshida, W.Y.; McClintock, J.B. Chemical and ecological studies of the Antarctic sponge *Dendrilla membranosa*. *J. Nat. Prod.* **1995**, *58*, 1459-1462.

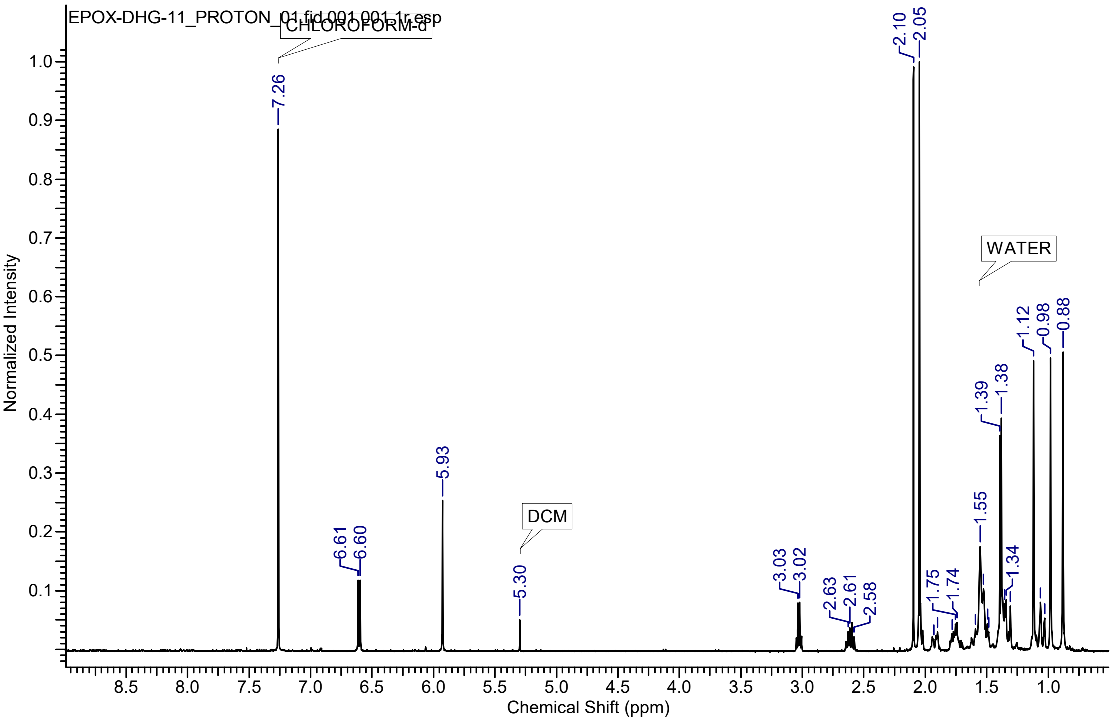

Figure S28. Dendrillin A (**9**) ^1H NMR spectrum (400 MHz, $CDCl_3$).

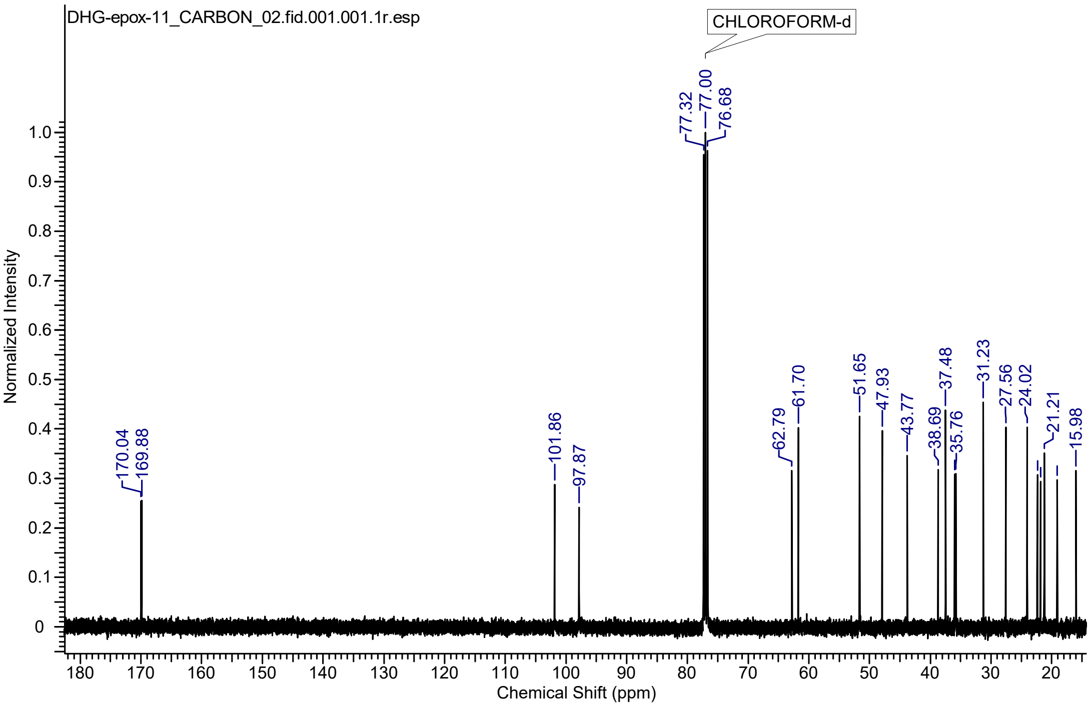

Figure S29. Dendrillin A (**9**) ^{13}C NMR spectrum (101 MHz, $CDCl_3$).

*Conversion of membranolide (**5**) to the corresponding triol (**15**):* A solution of membranolide (**5**) (25 mg, 0.073 mmol) in dry ether (4.0 mL) was added to a stirred suspension of lithium aluminum hydride (25 mg, 0.66 mmol) in dry ether (5.0 mL) under an atmosphere of dry nitrogen. The mixture was heated under reflux for 2 hr, cooled to 0 °C, and quenched with ethyl acetate, followed by 2 M hydrochloric acid (3 mL). The mixture was extracted with ethyl acetate (3 X 15 mL), and the combined organic extracts were washed with a dilute sodium bicarbonate solution (5 mL), dried over Na_2S0_4, and evaporated to give 32.5 mg of a crude oil. The oil was purified by NPHPLC using a gradient (hexane: EtOAc, (0:1 → 1:0, v/v)). The isolated fraction contained the desire triol as suggested by the 1H NMR but also some other compounds. Therefore, an extra analytical HPLC separation was required to give approx. 1 mg of the pure triol (0.9 mg, 0.003 mmol, 4%). HRESIMS *m/z* 343.224 $[M + Na]^+$ 343.224 calculated for $C_{20}H_{36}O_3Na$. ^{1}H NMR and ^{13}C NMR data see **Error! Reference source not found.**. Adapted from procedure reported in: Molinski, T.F.; Faulkner, D.J. Metabolites of the Antarctic sponge *Dendrilla membranosa*. *J. Org. Chem.* **1987**, *52*, 296-298.

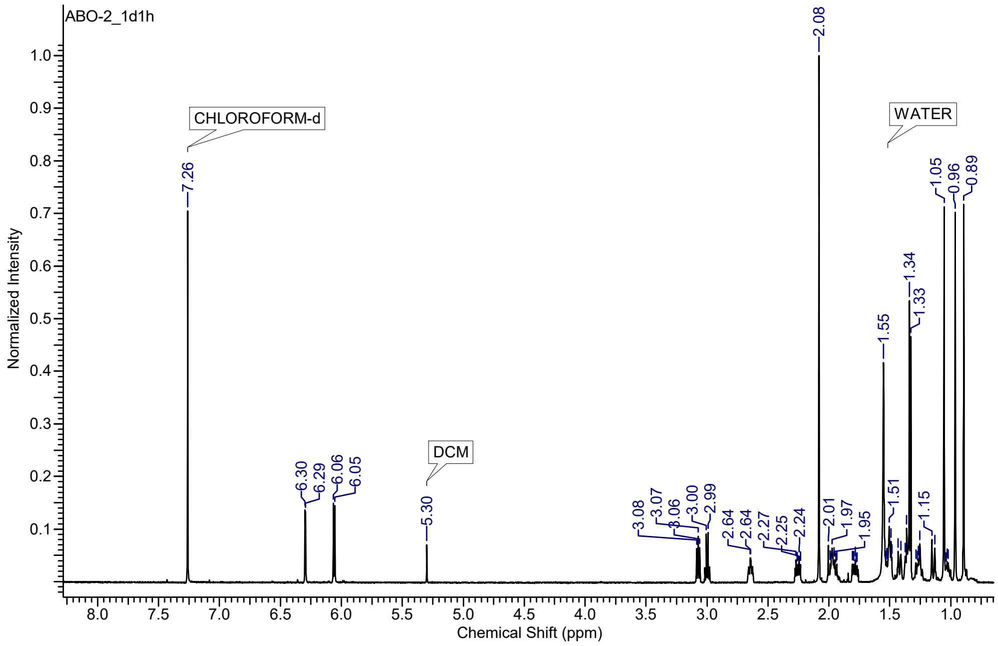

Figure S30: Triol **15** ^{1}H NMR spectrum (400 MHz, $CDCl_3$).
